# PRV-1 Virulence in Atlantic Salmon Is Affected by Host Genotype

**DOI:** 10.3390/v17020285

**Published:** 2025-02-19

**Authors:** Mark Polinski, Lynden Gross, David Groman, Marta Alarcón, Mark Braceland, Marije Booman, Delphine Ditlecadet, Samuel May, Nellie Gagné, Kyle Garver

**Affiliations:** 1 U.S. Department of Agriculture National Coldwater Marine Aquaculture Center, Orono, ME 04473, USA; samuel.may@usda.gov; 2 Fisheries and Oceans Canada Pacific Biological Station, Nanaimo, BC V9T6N7, Canada; lynden.gross@dfo-mpo.gc.ca; 3 University of Prince Edward Island Atlantic Veterinary College, Charlottetown, PEI C1A4P3, Canada; groman@upei.ca; 4 Pharmaq Analytiq, 5008 Bergen, Norway; marta.alarcon@zoetis.com; 5 Onda, Souris, PEI C0A2B0, Canada; mark.braceland@wellfishtech.com (M.B.); mbooman@onda.ca (M.B.); 6 Fisheries and Oceans Canada Gulf Fisheries Research Centre, Moncton, NB E1C5K4, Canada; delphine.ditlecadet@dfo-mpo.gc.ca (D.D.); nellie.gagne@dfo-mpo.gc.ca (N.G.)

**Keywords:** piscine orthoreovirus (PRV), atlantic salmon, virulence, laboratory disease challenge, genotype

## Abstract

Heart and skeletal muscle inflammation (HSMI) is a significant disease affecting Atlantic salmon (*Salmo salar*) production in Norway but has had limited impact to production in North America. The causative agent of HSMI is piscine orthoreovirus genotype 1 (PRV-1), and disease variation between regions is suggested to be at least partially driven by genetic variation of the virus. Using controlled laboratory injection challenges, we corroborate variations in disease outcomes for three PRV-1 isolates (PRV-1a from the eastern Pacific, PRV-1a from the western Atlantic, and PRV-1b from the Norwegian sea); however, virus replication dynamics, host recognition, and PRV-1-associated heart inflammation were also discrete relative to the Atlantic salmon stock challenged, irrespective of the viral isolate used. Specifically, New Brunswick Tobique River Atlantic salmon had less (*p* < 0.01) heart inflammation relative to Mowi-McConnell Atlantic salmon of Western Canada which, in turn, had less (*p* < 0.01) heart inflammation than Mowi Atlantic salmon of Scotland when cumulatively considering challenges using all three PRV-1 isolates. These data indicate that the presence of PRV-1a or PRV-1b alone is not sufficient to reliably predict disease and highlights at least one potential mechanism (host genotype) for reducing HSMI disease severity.

## 1. Introduction

The primary drivers of piscine orthoreovirus genogroup 1 (PRV-1) virulence are largely unknown. Despite the establishment of a causative link between PRV-1 and the disease of farmed Atlantic salmon known as heart and skeletal muscle inflammation (HSMI) [1], infection with PRV-1 does not always result in disease [2]. In fact, the virus is relatively commonplace across countries with Atlantic salmon farming, but the occurrence of HSMI is not [3]. For example, in Norway—where PRV-1 and HSMI were first identified [4,5,6]—PRV-1 is ubiquitous among farmed Atlantic salmon and HSMI poses a significant and widespread production concern for the industry (e.g., 150–180 farm-level HSMI cases per year across >600 active farms) [7]. In contrast, in Pacific Canada, PRV-1 is also ubiquitous across the Atlantic salmon industry, reaching high viral loads, while the occurrence of HSMI-like disease is rare (e.g., typically 0 or occasionally 1 farm-level HSMI-like case per year across >70 farms) [2,8,9,10]. Differential disease outcomes of PRV-1 infection have also been observed across controlled laboratory exposure studies with similar environmental parameters where the universal establishment of high load systemic infections have produced varying amounts of heart inflammation [1,2,11,12].

Current data indicate that PRV-1 virulence is at least partially driven by genetic variation within the virus. This is supported by a side-by-side controlled laboratory comparison of six PRV-1 genetically unique isolates which differed in their ability to induce HSMI-like heart inflammation in a European stock of Atlantic salmon [12]. Specifically, isolates categorized in the PRV-1a genotype based on S1 genome segment phylogeny were demonstrated to have lower virulence than two PRV-1b isolates, and this has been supported by HSMI field observations [13]. Additional consideration of other genome segments has further led to the hypothesis that viral reassortment of two ancestral genotypes in Norwegian aquaculture gave rise (and may continue to give rise) to virulent phenotype(s) of PRV-1, and that genotypic variation in segments beyond S1 are required for increased virulence [14,15]. One current unexplained caveat is that the PRV-1 genotype is not always explanatory of disease—as evidenced by occasional farm-level diagnoses of HSMI or HSMI-like disease with PRV-1 infections that are considered to be “low-virulence” genotypes [2,9,16].

Host and environmental factors also implicitly influence disease outcome; yet fewer data are available for implicating either host or environmental conditions in the establishment of HSMI. This disease appears to be associated with an adaptive cytotoxic T cell response by Atlantic salmon to PRV-1 antigen (reviewed by [3]) and, thus, modulation of salmon T cell responses would be expected to impact disease outcomes. This is supported by investigations involving the dietary modulation of Atlantic salmon immune responses that may at least partially mitigate HSMI [17,18,19]. Furthermore, some Atlantic salmon stocks may inherently be more (or less) predisposed to PRV associated disease, as suggested by the development of a strain of Atlantic Salmon in Norway that has been claimed to have less heart damage and higher survival in association with HSMI yet is equally susceptible to PRV-1 infection [20,21].

Laboratory vs. field observations for HSMI severity also clearly indicate an environmental component associated with this disease [3], yet the particular mechanisms in field environments that enhance HSMI outcomes are unknown. Although “stress” associated with production environments has been commonly hypothesized to contribute to HSMI, application of maximal glucocorticoid, forced transition to saltwater, or viral coinfection stressors in laboratory environments have been insufficient to recreate equivalent disease [11,22,23].

In this study, we aimed to investigate the degree to which host genetic differences affect PRV-1-associated disease and compare the influence of host Atlantic salmon strain directly to PRV-1 genotype to see which factor had a greater impact on PRV-1-associated disease. We hypothesized that both the host strain of Atlantic salmon and the viral genotype would influence disease severity following the disease triangle principle [24], but that viral genotype would have a greater influence based on currently available data that suggest at least some PRV-1b isolates appear to be more strongly associated with disease in both the laboratory and field.

## 2. Materials and Methods

### 2.1. PRV-1 Isolates

Three PRV-1 isolates were used in this study: BC-16-005ND, NOR-2018NL, and NB-2018-128 (Table 1). The first isolate, BC-16-005ND, originated from a commercial farmed Atlantic salmon from the Pacific coast of British Columbia, Canada [2]. Viral particles for this isolate were purified from infected whole blood as previously described [1]. Briefly, sonicated and clarified blood homogenate was combined with Vertrel XF (DuPont Chemicals, Wilmington, DE, USA) was layered onto a 1.22–1.45 g/mL cesium chloride gradient and centrifuged using a SW 40 TI rotor in an Optima LE 80k Ultracentrifuge (Beckman Coulter, Brea, CA, USA) at 40,000 rpm for 20 h. Viral particles were collected from the 1.22/1.45 interphase by needle puncture and dialyzed at 4 °C against fresh Dulbecco’s PBS for 1, 3, and >12 h using a 7K Slide-A-Lyzer Dialysis Cassette (Thermo Fisher, Waltham, MA, USA) before 15% glycerol was added prior to freezing and storage at −80 °C. The next isolate, NOR-2018NL, was obtained as cesium chloride-purified particles from the Norwegian University of Life Sciences with demonstrated virulent potential in experimental challenge trials [12]. The third isolate, NB-2018-128, was obtained from a commercially farmed Atlantic salmon in New Brunswick, passed through Atlantic salmon (n = 6) by intraperitoneal injection of a 0.1 mL homogenized blood cell pellet, and harvested from whole blood via needle puncture of the caudal vein 6 weeks later. Although cesium chloride purification methods were attempted for NB-2018-128, the low viral load (as measured by qPCR) resulted in insufficient recovery of purified particles to conduct subsequent challenge trials. Therefore, sonicated and low-speed centrifuge-clarified blood homogenate diluted in PBS was used directly as the inoculate for challenge trials for this isolate, as previously described [2]. For each PRV-1 isolate, a 10 µL sample immediately prior to challenge was used to quantify genomic material by qPCR (see Section 2.4). All viral isolates were stored at −80 °C prior to use.

Challenge inoculates were prepared the day of challenge from either cesium chloride-purified (BC-16-005ND—Challenge 1 and 2; NOR-2018NL—Challenge 2) or clarified blood homogenate (NB-2018-128, Challenge 2) frozen stocks by diluting each to a final concentration of 1 × 10^8^ PRV-1 genome copies per mL in refrigerated phosphate-buffered saline with a final concentration of 0.01% glycerol. Control inoculate was identically prepared without virus. For clarified blood homogenate inoculate (NB-2018-128), blood homogenate made up less than 10% (10 uL) of the total volume.

### 2.2. Atlantic Salmon—Sources and Husbandry

Atlantic salmon were sourced from four independent hatcheries representing four regionally distinct genetic strains that generally encompass the major stocks used in commercial production globally (Table 1). All fish were transported by 2-day courier directly to Onda, in Souris, Prince Edward Island. One strain was sourced as ~10 g parr from a commercial hatchery on Vancouver Island, British Columbia. These Atlantic salmon were of a Pacific-adapted Mowi-McConnell strain from British Columbia with at least 30 years isolation from the originating European stocks [25]. The second stock was sourced as ~10 g parr from a commercial hatchery in New Brunswick, Canada, of a St. John River strain that have been selectively bred for aquaculture production for more than 16 years [26]. The third stock of fish was obtained as ~10 g parr from Mactaquac Biodiversity Facility, New Brunswick, Canada, where hatchery-spawned wild-caught salmon from the Tobique River [27] were manually crossed to produce the F1 generation used in this study. The fourth stock of fish was obtained as eggs from a commercial aquaculture facility in Scotland, UK, rearing the domesticated Mowi Atlantic salmon strain heavily used in Norway and Scotland for commercial production. All stocks were of a mixed-sex, mixed-family composition.

Once at Onda, fish were maintained in UV-treated flow-through fresh well water (7–12 °C) for 3–6 months. Upon reaching approximately 40–50 g mean body weight, each stock was progressively acclimated over a 20-day period to UV-irradiated brackish well water (10–12 °C, 25 ppt NaCl). A 12 h light–12 h dark photoperiod was used except during saltwater acclimation, when a 24 h light cycle was temporarily employed. Fish were fed dry pellets (EWOS) at 2–5% body weight per day prior to the challenge. During both challenge trials, fish were maintained in UV-irradiated brackish well water (11 ± 1 °C, 25 ppt) and fed a ration of EWOS pellets at 1% body weight per day in biosafety level 3 (BSL-3) containment.

### 2.3. PRV-1 Challenges of Atlantic Salmon

Two challenge trials were conducted as part of this study. Challenge 1 utilized a single PRV-1 isolate (BC-16-005ND hereafter referred to as BC-PRV) administered to two strains of Atlantic salmon: BC Mowi-McConnell (BC salmon; mean weight 43.7 g) and New Brunswick St. John River (NB-SJR salmon; mean weight 43.3 g) (Table 1). An intraperitoneal injection of 100 μL was administered individually to 50 fish per tank at a targeted dose of 1 × 10^6^ BC-PRV reverse-transcribed L1 genomic copies per 100 μL inoculate to duplicate tanks of either BC or NB-SJR salmon. Inoculate without virus was identically prepared and administered to duplicated tanks for each salmon strain to act as vehicle saline control (SC). All fish were anesthetized in 75 mg per L tricaine mesylate (MS-222) prior to intraperitoneal injection. Following injection, 6 fish per treatment from one replicate tank were immediately sampled following an overdose of MS-222 (200 mg per L). Subsequently, every two weeks post-challenge (wpc) 6 fish per treatment were sampled for a period totaling 14 weeks. Whole blood (100 μL) was collected via caudal vein puncture and immediately frozen at −80 °C for PRV screening and host gene expression analysis by qPCR. Hearts were bisected longitudinally, and one half preserved in 10% neutral buffered formalin for histopathology while the other half was immediately frozen at −80 °C for PRV screening and host gene expression (qPCR) analyses.

Challenge 2 utilized three PRV-1 isolates—BC-16-005ND (BC-PRV), NOR-2018NL (NOR-PRV), and NB-2018-128 (NB-PRV)—that were administered to three strains of Atlantic salmon: BC Mowi-McConnell (BC salmon; mean weight 59.5 g), New Brunswick Tobique River (NB-TR salmon; mean weight 44.5 g), and European Mowi (EU salmon; mean weight 70.7 g) from Scotland (Table 1). An intraperitoneal injection of 100 μL inoculate was administered individually to 50 fish per tank at a targeted dose of 1 × 10^6^ PRV-1 reverse-transcribed L1 genomic copies per 100 μL to duplicate tanks of each salmon strain. Inoculate without virus was identically prepared and administered to duplicate tanks of each salmon strain to act as the vehicle saline control (SC). As per Challenge 1, fish were anesthetized in 75 mg per L MS-222 prior to intraperitoneal injection. Immediately following injection, four fish per treatment were lethally sampled (two from each duplicate tank) and every 2 wpc, eight fish per treatment were lethally sampled (four from each duplicate tank) for a period totaling 14 weeks. A portion of whole blood (100 μL) was collected via caudal vein puncture and immediately frozen at −80 °C for PRV screening by qPCR. A second portion of whole blood (up to 900 μL) was collected and spun at 2000× *g* for 10 min at 4 °C in a lithium–heparin-treated vacutainer (BD) from which 100 μL plasma was transferred to a fresh 1.5 mL tube and frozen at −80 °C for PRV screening. Approximately 200 mg section of red/white skeletal muscle was excised from the left lateral line at approximately the mid-body and preserved in 10% neutral buffered formalin (NBF). Hearts were bisected longitudinally, and one half was preserved in 10% neutral buffered formalin while the other half was immediately frozen at −80 °C for qPCR analyses. Inventories of the analyzed data for Challenge 1 and Challenge 2 are presented in Tables S1 and S2, respectively.

### 2.4. PRV-1 Detection and Quantification

PRV-1 L1 RNA was detected in whole blood, plasma, heart, and inoculate samples by real-time quantitative PCR (qPCR) following reverse transcription at the Fisheries and Oceans Canada Pacific Biological Station in Nanaimo, British Columbia, based on previously published methods [8]. Specifically, total RNA was extracted from 100 μL blood, approximately 50 mg tissues, or 10 μL purified viral inoculates in TRIzol Reagent (Life Technologies, Carlsbad, CA, USA) as per the manufacturer’s instructions that implemented a 5 mm steel bead and TissueLyser II (Qiagen, Germantown, MD, USA) operating for 2 min (blood) or 4 min (heart) at 25 Hz. Nucleic acids were extracted from 10 μL of plasma diluted in 130 μL PBS using the QIAamp Viral RNA Mini Kit (Qiagen). A portion of eluted RNA (1.0 μg from whole blood or tissue; 10 μL for plasma and inoculate) was denatured for 5 min at 95 °C and immediately cooled to 4 °C. Denatured blood, tissue, and viral inoculate RNA were reverse transcribed using a high-capacity cDNA reverse transcription kit (Life Technologies) following the manufacturer’s instructions. The resulting cDNA was used directly as a template for qPCR analysis in a StepOne-Plus real-time detection system (Applied Biosystems, Waltham, MA, USA) using previously described primers and TaqMan probe [4]. Each reaction contained 400 nM primers and 300 nM TaqMan probe, 1X TaqMan Universal Master Mix, and 1 μL cDNA template within each 15 μL reaction. Cycling conditions included an initial incubation of 95 °C for 10 min followed by 40 cycles of 95 °C for 10 s and 60 °C for 30 s. Denatured plasma RNA (5 μL) was reverse transcribed and assayed with the OneStep RT-PCR Kit (Qiagen) following the manufacturers protocol and using the same concentration of PRV L1 primers and TaqMan probe specified above, 1× OneStep RT-PCR Buffer, dNTP Mix (400 μM of each dNTP), 1× Q-Solution, 500 nM ROX reference dye, and 1 μL OneStep RT-PCR Enzyme Mix, within each 25 μL reaction. Reverse transcription and cycling occurred at 50 °C for 30 min and 95 °C for 15 min, with 40 cycles of 94 °C for 15 s, followed by 60 °C for 30 s and 72 °C for 30 s. All samples were assayed in duplicate and were considered positive if both technical replicates reported a Ct value  <  40 cycles or negative if one or both technical replicates failed to fluoresce beyond the preset threshold (∆ Rn 0.01) during 40 cycles. PRV L1 RNA quantification was determined in each positive instance by serial dilution of a 482 bp double-stranded DNA gBLOCK fragment (Integrated DNA Technologies, Coralville, IA, USA) consisting of sequence targeted by the qPCR primer and probe [11] using a seven-step tenfold dilution series of the gBLOCK fragment spanning a dynamic range of 10–10^7^ target copies per reaction.

### 2.5. Atlantic Salmon Gene Expression and Histopathology

Histopathology was used in this study to measure inflammatory disease to coincide with how HSMI is diagnosed in the field [28,29]. Tissues in 10% NBF were fixed for 24–48 h, transferred to 70% isopropanol, and paraffin-embedded following standard methods. Sections 3 µm thick were transferred to glass slides and stained routinely with hematoxylin and eosin for light microscopy. For Challenge 1, a single pathologist (Michael Pawlik, BC Ministry of Agriculture and Food) reviewed all histopathology samples following a previously published scoring metric ([2]; Table S1). For Challenge 2, a single pathologist (David Groman, University of Prince Edward Island) reviewed all histology samples following a slightly different published scoring metric ([29]; Table S2). To ensure inter-sample consistency as well as consistency relative to other published PRV studies, approximately 20% of samples collected as part of Challenge 2 (144 of 713) were also reviewed by a second pathologist (Marta Alarcón, Pharmaq Analytiq) with experience assessing HSMI in Norway (Table S2; Figure S1). All pathologists were blinded to the PRV exposure status, salmon stock, and scores provided by the other pathologist until after all scoring had been completed. Images of some heart tissues with associated scoring are provided in Figure S1.

For gene expression in Challenge 1, a portion (5 μg) of total RNA extracted from the blood and heart that was not reverse transcribed for the detection of the virus was purified using 2 U of DNase I (RNase free) (Life Technologies) at 37 °C for 45 min followed by RNeasy MinElute Cleanup (Qiagen) as per the manufacturer’s instructions. For Challenge 2, a portion (10 μg) of total RNA from the heart samples was subjected to DNase treatment (Qiagen) at ambient temperature for 10 min and column-purified with the RNeasy Mini Kit (Qiagen) following the manufacturer’s RNA Cleanup protocol. For both challenge trials, RNA (1.5 μg) was reverse transcribed using a high-capacity cDNA reverse transcription kit without RNase inhibitor in which the random primer mix was substituted with 5 μM Oligo d(T)16. All real-time qPCR analyses were conducted on a StepOne-Plus real-time detection system using SYBR green chemistry. Each reaction consisted of 1× Power SYBR master mix (Applied Biosystems), forward and reverse primers (800 nM each; [23]), and 1–2 μL cDNA template, to a final volume of 15 μL. Samples were assayed in duplicate with a five-step, fourfold dilution series of pooled cDNA included in each run to calculate amplification efficiency, linearity, and relative quantity, and to provide inter-run calibration. The cycling conditions consisted of an initial activation of DNA polymerase at 95 °C for 10 min, followed by 40 cycles of 95 °C for 5 s, 60 °C for 20 s, and 72 °C for 10 s. A melt-curve analysis was conducted for each run to ensure amplification specificity, and amplification efficiency for all assays was ensured to be greater than 90%. Five mRNA transcripts of immune-associated proteins were assessed for expression in this study. In Challenge 1, the *mx1* transcript which codes Myxovirus protein-1 was used to identify general viral recognition by host cells via activation of the type-1 interferon pathway. The *cd8a* and *gzma* transcripts code for the cluster of differentiation-8 protein and granzyme protein, respectively, and provided markers for CD8+ cytotoxic T cell occurrence and activation used in the host immune-directed killing of virus-infected cells. In Challenge 2, *cd8a* was again used to identify CD8+ cytotoxic T cell occurrence, while *ifna* and *ifng* transcripts which code for type-1 and type-2 interferon, respectively, were measured to differentiate general host cell recognition of the virus (type-1 interferon) relative to the professional immune cell recognition of the viral infection (type-2 interferon). All gene expression data were normalized to *actb* transcription that codes for the beta-actin protein which has previously shown stable expression following the PRV infection of salmon [22].

### 2.6. PRV-1 and Atlantic Salmon Genotyping

For PRV-1 genotyping, a portion (1 µg) of purified total RNA from 3 infected challenged fish (#176, #199, and #219) not used for qPCR analysis was pre-amplified using primer pairs designed in Primal Scheme (http://primalscheme.com; accessed on 18 February 2025) to target each of PRV-1′s 10 genomic segments (Table S3) and run in multiplex PCR, then cleaned and sequenced following previously published protocols [30] using a nanopore MinION sequencer. Reads were assembled against the published PRV-1 genome (GenBank GCA_002829625.1; [31]) using NanoPipe [32].

For Atlantic salmon genotyping, fin clips from 16 fish per stock during Challenge 2 were collected and preserved in ethanol—representing BC salmon, EU salmon, and NB-TR salmon. NB-SJR and BC salmon used in Challenge 1 were not fin clipped at the time of sampling; however, BC stocks were sourced from the same hatchery and breeding program as for Challenge 2 (only 1 cohort earlier), and 16 fin clips from SJR stocks held at the U.S. Department of Agriculture National Coldwater Marine Aquaculture Center in Franklin, ME, from the same cohort year and originating breeding program as those used in Challenge 1 were genotyped as representative proxies. All fin clips were sent to the Center of Aquaculture Technologies (San Diego, CA, USA) for DNA extraction and single nucleotide polymorphism (SNP) genotyping using a recently developed 50 k SNP array for North American Atlantic salmon ([33]; publicly available via Thermo Fisher Axiom USDA Sal, SKU# 551,627 and 551628). The resulting SNP microarray dataset was filtered using the adegenet package [34] in R (v.4.3.1; R Core Team). To ensure high-quality genotype data, loci were removed if genotyped in <90% of individuals. No individuals were genotyped at <90% of loci and, thus, they were not filtered. Monomorphic loci or SNPs with a minor allele frequency (MAF) ≤ 0.05 were removed to eliminate rare variants or possible genotyping errors. To remove non-neutral loci that were putatively under selection, exhibiting non-Mendelian inheritance, or possible null alleles, loci that significantly deviated from the Hardy–Weinberg equilibrium (HWE, estimated in pegas, v.0.13; [35]) or with an absolute difference between expected and observed heterozygosity (|He-Ho|) > 0.2 in any one of the four populations were removed. These filtering steps ensured that the retained loci were robust for neutral population genetic analyses.

### 2.7. Statistical Analyses

Phylogenetic comparison of concatenated segments of the three PRV-1 isolates used in this study was performed against 48 published whole genome sequences, as presented by Siah et al. [36] via a Tamura–Nei neighbor-joining method following Clustal Omega maximum-likelihood alignment in Geneious Prime 2024.0.5 using 1000 bootstrap iterations. Genetic differentiation among the four populations of Atlantic salmon was assessed using Weir and Cockerham’s Fst, estimated in the hierfstat package [37]. Confidence intervals for Fst values were determined via non-parametric bootstrapping of loci with 1000 iterations. To visualize genetic variation among populations, a genetic principal component analysis (PCA) was implemented in adegenet. As this analysis does not tolerate missing data, missing genotypes were imputed using population medians.

For the Challenge 1 experiment, BC-PRV transcriptional blood load as well as fold change in Atlantic salmon *mx1*, *cd8a*, and *gzma* relative to mean SC timepoint- and salmon strain-matched expression in blood and heart was compared via two-way ANOVA, followed by Sidak’s multiple comparison tests of log-transformed data. Categorical heart inflammatory scores for epicarditis and endocarditis were compared separately between treatments using a Kruskal–Wallis test followed by Dunn’s multiple comparison tests.

For the Challenge 2 viral load comparisons, PRV-1 L1 RNA quantities were assessed at both the peak and persistent phases of infection by a one-way ANOVA and Tukey’s multiple comparison tests of log-transformed quantities. As peak infections occurred at slightly different time points depending on the PRV-1 isolate × Atlantic salmon strain challenge (Figure S2), we considered the eight highest recorded values obtained between 6–10 wpc from each PRV-1 isolate × Atlantic salmon strain combination as being representative of the peak infection and categorized these data for comparison either by the PRV-1 isolate received (n = 24 per category) or by the Atlantic salmon strain challenged (n = 24 per category). Persistent infections were assessed at 14 wpc for all PRV-1 isolate × Atlantic salmon strain challenges (n = 8 per specific challenge) categorized in the same manner—either by PRV-1 isolate (n = 24) or Atlantic salmon strain (n = 24).

For the Challenge 2 heart *cd8a*, *ifna*, and *ifng* gene expression analyses, quantities of each target transcript were normalized to *actb*—a transcript previously demonstrated to have stable expression following PRV infection of salmon [11,22]—and scaled to the minimum observed value (i.e., the corrected normalized relative quantity; CNRQ). A Pearson r correlation was obtained for *cd8a* and *ifna* or *ifng* based on log-transformed values from the full dataset (n = 384 samples obtained between 6–12 wpc). The CNRQ of *cd8a* and *ifna* expression in relation to histopathological cardiac inflammation score was also assessed for the complete data set using the Kruskal–Wallis nonparametric test and Dunn’s multiple comparison tests based on inflammation score. The CNRQs of *cd8a* transcripts from the entire data set sans SC samples (n = 288) were compared by two-way ANOVA and Šídák’s multiple comparison tests of log-transformed CNRQ values categorized either by PRV-1 isolate (n = 24 per PRV-1 genotype per time point) or by Atlantic salmon strain (n = 24 per salmon genotype per time point).

For Challenge 2 histopathological observations, cumulative categorical heart inflammatory scores of PRV-1-challenged individuals between 2–14 wpc (n = 504) were categorized by either PRV-1 isolate (BC, NB, or NOR) or Atlantic salmon strain (BC, NB-TR or EU) and compared using the Kruskal–Wallis nonparametric test and Dunn’s multiple comparison tests. All comparisons were performed using GraphPad Prism 10.3.0. As principal component analysis and direct variance comparisons suggested homogeneity between tank replicates in this study, samples collected from replicate treatment tanks were pooled for all the analyses described above.

## 3. Results

### 3.1. Phylogentic Variation in Atantic Salmon Strains and PRV-1 Isolates

**Figure 1:**
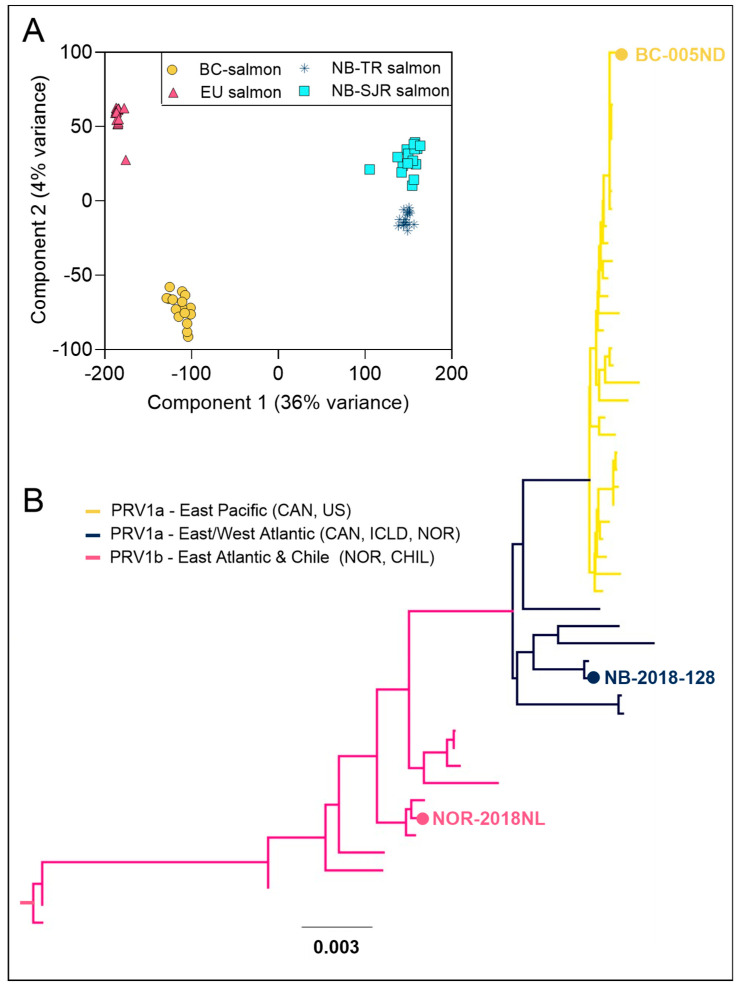
(**A**) Two-dimensional variance determined by a genetic principal component analysis of 50 k microarray genotypes among individual Atlantic salmon (n = 64) representing 4 geographically separate stains: British Columbia, Canada—Mowi-McConnell (BC salmon), European (Scotland) origin Mowi (EU salmon), New Brunswick, Canada—Tobique River (NB-TR salmon), and New Brunswick, Canada—Saint John River (NB-SJR salmon). (**B**) Tamura–Nei neighbor-joining phylogram indicating the genetic diversity of 48 concatenated PRV-1 genomes as presented by Siah et al. [36], as well as the 3 concatenated PRV-1 genomes sequenced in this study (highlighted with text) following Clustal Omega maximum-likelihood alignment. Phylogenetic groups (PRV-1b from Eastern Atlantic and Chile, PRV-1a from both Western and Eastern Atlantic, and PRV-1a from the Eastern Pacific) are indicated by branch lines color at 100% bootstrap consensus support. Scale bar indicates the genetic divergence as the average nucleotide substitutions per position.

Unique genetic structuring was observed for each Atlantic salmon strain used in this study. Filtering processes for the microarray panel data reduced the number of SNP loci from 55,044 to 32,381 SNPs. The retained loci were used to estimate pairwise Fst values among the four populations (Table S4), confirming varying degrees of genetic differentiation between populations and subspecies. Principal component analysis also demonstrated clear separation between the four populations (Figure 1A), supporting the Fst results. The first two principal components explained a substantial proportion of the genetic variance (35.71% and 3.77%, respectively), with clear clustering corresponding to each population, indicating distinct genetic structuring between the Atlantic salmon strains.

Unique genetic structuring was also observed for each PRV-1 isolate used in this study. A whole concatenated genome comparison of the three PRV-1 isolates from this study to the 48 PRV-1 genomes considered by Siah et al., 2020 [36], identified similar phylogenetic ordination using Tamura–Nei neighbor-joining methods to the Bayesian phylogenetic analyses previously employed. The BC-PRV grouped together with all other Northeastern Pacific PRV-1 isolates to form a monophyletic clade, NB-PRV grouped with other isolates from the East/West Atlantic, and NOR-PRV grouped with a subset of other Eastern Atlantic isolates from Norway and Chile (Figure 1B). Specific to the S1 component of these genomes, both BC- and NB-PRV were members of what has been previously designated as the PRV-1a group, while the NOR-PRV is within the PRV-1b group [31] shown to be associated with higher virulence in Norway [12]. Further virulence characterization of these isolates, as described by Vatne et al., 2021 [15] using 4 viral genomic segments (L1, L2, S1, and S4), classifies BC-PRV and NB-PRV within the “Low-2” and NOR-PRV within “High-1” genogroups. NCBI accession numbers for the PRV-1 sequences obtained in this study are shown in Table S5.

### 3.2. Challenge 1: PRV-1 Infection Dynamics and Host T Cell Responsiveness Can Vary Among Atlantic Salmon Strains

**Figure 2:**
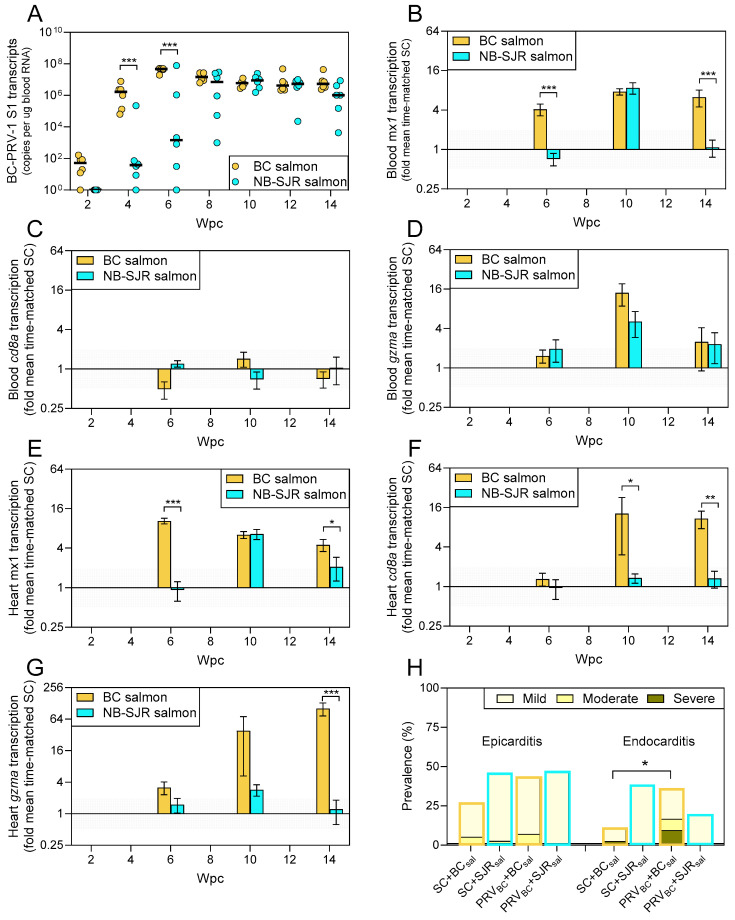
PRV-1 load, antiviral responsiveness, and inflammation in side-by-side challenge of BC Mowi-McConnell (BC) and New Brunswick St. John River (NB-SJR) Atlantic salmon. (**A**) Mean (line) and individual (dot) BC-PRV-1a L1 RNA loads measured 2–14 weeks post-challenge (wpc). Mean fold change (±SE) of (**B**) blood *mx1*, (**C**) blood *cd8a*, (**D**) blood *gzma*, (**E**) heart *mx1*, (**F**) heart *cd8a*, and (**G**) heart *gzma* transcripts relative to the mean of time-matched, strain-matched controls (SC); * *p* < 0.05, ** *p* < 0.01, *** *p* < 0.001 by two-way ANOVA and Šídák’s multiple comparisons tests of log-transformed fold changes; minimum twofold change suggestive of biological relevance is shaded. (**H**) The cumulative prevalence of epicarditis and endocarditis in hearts of control (SC) and BC-PRV-1a challenged (PRV) fish within 14 wpc. * *p* < 0.05 by the Kruskal–Wallis and Dunn’s multiple comparisons tests.

Identical aliquots of BC-PRV administered to BC and NB-SJR salmon in Challenge 1 produced different viral infection dynamics as well as host immune responsiveness in the two salmon strains. The BC-PRV took longer (by 4 weeks) to reach peak transcriptional loads in whole blood of NB-SJR fish (median 8.9 × 10^6^ at 10 wpc) than in BC fish (median 4.7 × 10^7^ at 6 weeks), with lower viral RNA blood loads at both 4 and 6 wpc for most NB-SJR individuals (Figure 2A). Not only did BC-PRV amplification appear to be delayed in NB-SJR salmon, but peak viral blood loads were also slightly lower than in BC salmon (mean 42% ± 19%, *p* = 0.009) when comparing the highest observed blood infections (n = 8) from each strain. Nevertheless, at peak replication, high loads were reached in nearly all individuals sampled in both salmon strains (mean 4.4 and 2.5 × 10^7^ copies/ug blood RNA, respectively). Persistent blood loads at 12 and 14 wpc also remained substantial in both salmon strains (mean > 1 × 10^6^ copies/ug blood RNA) (Figure 2A).

Systemic BC-PRV recognition as measured by whole blood *mx1* transcription—a marker for interferon pathway activation—became elevated (4–8-fold) in both BC and NB-SJR salmon, although this activation was considerably more prolonged in BC salmon (observed 6–14 wpc) relative to NB-SJR salmon (observed only at 10 wpc; Figure 2B). Whole blood transcription of *cd8*– a transcript marker for cytotoxic T cell presence, differentiation, and activation—did not appear to be affected by the presence of BC-PRV (Figure 2C), although *gzma*—a transcript associated with cytotoxic T cell-directed killing—was similarly elevated by 5–14-fold at 10 wpc in both salmon strains (Figure 2D). Together, these data suggest that BC-PRV was systemically recognized in blood by infected host cells (although for a longer period in BC salmon) with transient low-level increases in cytotoxic T cell killing activity, but without evidence of increased cytotoxic T cell presence.

In the heart, *mx1* transcriptional patterns mirrored those observed within whole blood, with 4–10-fold increases in transcription occurring in both salmon strains with more prolonged activation in BC salmon (Figure 2E). However, in contrast to *cd8* and *gzma* expression in the blood, heart expression of these transcripts was highly salmon strain-dependent. Transcription of both *cd8* and *gzma* were unaffected by BC-PRV in hearts of NB-SJR salmon, whereas mean *cd8* transcription became elevated 10–12-fold in hearts of BC salmon at 10–14 wpc (Figure 2F) and mean *gzma* expression was increased by approximately 100-fold at 14 wpc in BC salmon relative to controls (Figure 2G). Additionally, cumulative heart endocarditis (but not epicarditis) was elevated in BC-PRV-infected BC salmon, but not in BC-PRV-infected NB-SJR salmon relative to strain-matched SC fish (Figure 2H). These data indicate that although BC-PRV was recognized in infected hearts of both salmon genotypes, cytotoxic T cell presence and/or differentiation as well as activation of T cell-specific killing pathways were enhanced in BC salmon and not in NB-SJR salmon, and this T-cell activation in BC salmon was associated with elevated endocarditis.

### 3.3. Challenge 2: PRV-1 Infection Dynamics Can Be Affected More by PRV-1 Isolate than Atlantic Salmon Strain

**Figure 3:**
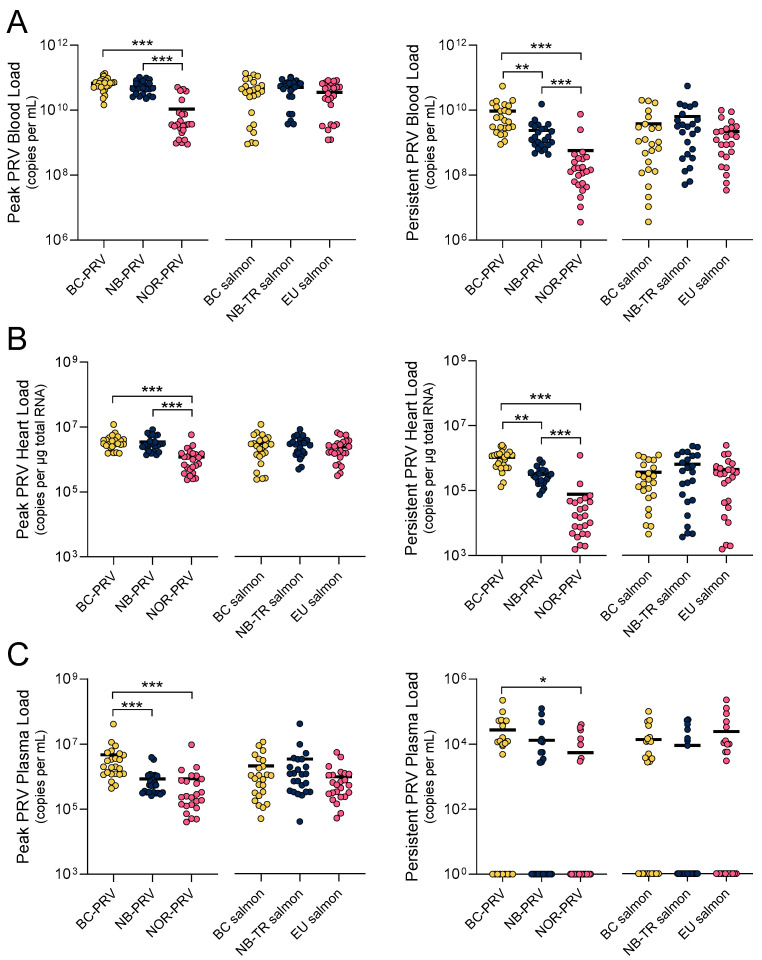
PRV-1 RNA loads of 72 individuals experiencing peak (6–10 wpc; left panels) or persistent (14 wpc; right panels) infections, categorized by either the strain of Atlantic salmon challenged (right component each panel) or the PRV-1 isolate administered (left component each panel). Mean (line) and individual (dot) PRV-1 L1 RNA loads identified in (**A**) whole blood, (**B**) the heart ventricle, or (**C**) plasma presented as the peak (eight highest recorded values 6–10 wpc from each salmon-virus challenge combination; n = 24 per category) or persistent (eight recorded values 14 wpc from each salmon-virus challenge combination; n = 24 per category) phases of infection. * *p* < 0.05, ** *p* < 0.01, *** *p* < 0.001 by one-way ANOVA and Tukey’s multiple comparisons tests of log-transformed values.

PRV-1 infection dynamics can be generally categorized into three temporal phases: early entry and dissemination, peak systemic replication, and long-term persistence [3]. We confirmed this general temporal pattern of systemic viral load in all challenges performed in this study, although the exact timing of each phase slightly varied based on host–virus combinations (Figure S2). Therefore, we assessed 72 individuals at both the peak (6–10 wpc depending on the PRV-1 isolate × salmon strain combination) and persistent (14 wpc for all PRV-1 × salmon combinations) phases of infection and compared these individuals based either on the PRV-1 isolate they were challenged with or by which salmon strain they belonged to. This revealed that the peak PRV-1 L1 RNA load in whole blood—a presumptive measure for intracellular systemic PRV-1 replication—was similar in fish administered with either BC- or NB-PRV genotypes (mean peak blood loads of 6.7 ± 0.6 SE and 5.3 ± 0.5 SE × 10^11^ copies/mL, respectively; *p* = 0.59), but was 4–5 times lower for fish administered NOR-PRV (1.1 ± 0.3 SE × 10^11^ copies/mL; *p* < 0.0001) when assessed cumulatively across the three salmon strains (Figure 3A). These quantitative relationships were mirrored in heart tissues, but at universally lower concentrations (Figure 3B).

By the persistent phase of infection, salmon injected with BC-PRV maintained the highest blood loads (9.3 ± 2.3 SE × 10^10^ copies/mL; *p* < 0.002), followed by fish injected with NB-PRV (2.4 ± 0.6 SE × 10^10^ copies/mL; *p* < 0.002) and then those injected with NOR-PRV (0.6 ± 0.3 SE × 10^10^ copies/mL; *p* < 0.0001) (Figure 3A). These quantitative relationships were also mirrored in heart tissue at universally lower concentrations (Figure 3A,B).

Specific to the plasma component of blood—a presumptive measure for systemically shed virus particles—peak PRV-1 RNA loads were highest for BC-PRV (4.8 ± 1.7 SE × 10^6^ copies/mL; *p* < 0.001) relative to either NB-PRV- or NOR-PRV-injected individuals, which were similar (8.5 ± 1.9 SE × 10^5^ copies/mL and 8.7 ± 4.0 SE × 10^5^ copies/mL, respectively; *p* = 0.09) (Figure 3C). The maintenance of higher BC-PRV plasma loads (at least relative to NOR PRV) was putatively suggested in the persistent phase of infection as well (*p* < 0.03); however, non-detections and relatively low quantities of RNA were observed for all three PRV-1 isolates, suggesting that these putative variations may be of limited biological relevance (Figure 3C).

In considering Atlantic salmon strain rather than PRV-1 isolate in these challenges, similar PRV-1 loads were recorded between the three salmon strains in the whole blood, heart, and plasma samples, at peak and persistent phases of infection (Figure 3). Cumulatively, these data suggest that PRV-1 isolate had somewhat unique infection dynamics in this study, whereby BC-PRV and to a more limited extent NB-PRV showed a higher capacity for viral replication, dissemination, and persistence than NOR-PRV. Conversely, these data demonstrated that variations in host Atlantic salmon strain had limited to no impact on PRV-1’s capacity for replication, dissemination, and persistence.

### 3.4. Challenge 2: Atlantic Salmon T Cell Responsiveness Can Be Affected by Both PRV-1 Isolate and Atlantic Salmon Strain

**Figure 4:**
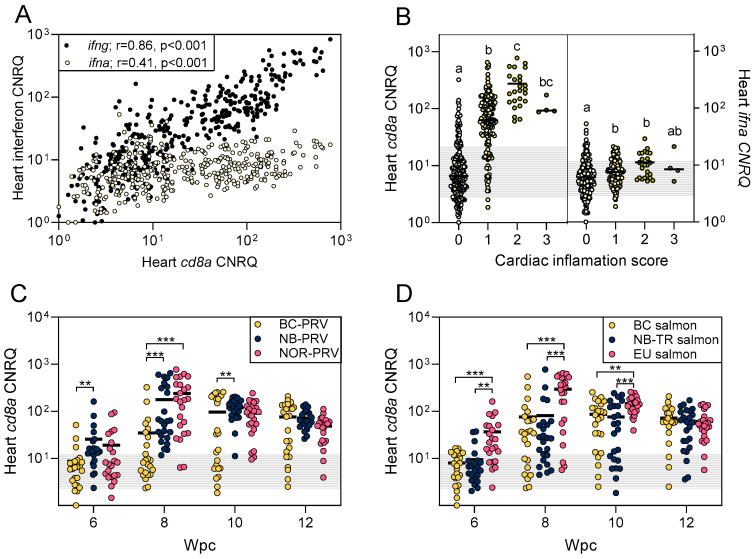
Heart *cd8a, ifna*, and *ifng* transcriptional expression following PRV-1 challenge. (**A**) Corrected normalized relative quantity (CNRQ) of *ifna* (light circles) and *ifng* (dark circles) are presented relative to *cd8a* transcriptional expression for all (n = 384) salmon sampled between 6–12 wpc in this study. Pearson r (r) and associated *p*-value is provided. (**B**) Mean (line) and individual (dots) heart *cd8a* (left side) and *ifna* (right side) CNRQ transcripts in relation to histopathological cardiac inflammation score, where 0 = no inflammation (n = 201), 1 = mild inflammation (n = 151), 2 = moderate inflammation (n = 25), and 3 = severe inflammation (n = 4) for the same 6–12 wpc dataset. Letters denote groupings for mean statistical similarity by the Kruskal–Wallis nonparametric test and Dunn’s multiple comparisons tests at *p* < 0.01. Shaded areas present one standard deviation of target transcription in cardiac inflammation score 0 fish to provide a minimum threshold suggestive of biological relevance. (**C**) Mean (line) and individual (dots) of heart *cd8a* transcripts measured at 6–12 wpc from PRV-1-challenged salmon (n = 288) categorized relative to PRV-1 isolate administered (n = 24 per PRV-1 isolate per time point), or (**D**) relative to Atlantic salmon strain challenged (n = 24 per salmon strain per time point). ** *p* < 0.01, *** *p* < 0.001 by two-way ANOVA and Šídák’s multiple comparisons tests of log-transformed CNRQ values; one standard deviation of *cd8a* expression recorded across all SC fish (n = 96) is shaded to suggest a minimum threshold for biological relevance.

A previous study has indicated that activation of CD8+ cytotoxic T cells is associated with heart inflammation during PRV-1 infections (reviewed by [3]). Here, we measured the transcription of *cd8*—a transcript marker for cytotoxic T cell presence, differentiation, and activation—along with *ifng*—a transcript marker for cytotoxic T cell and/or natural killer cell activation—and *infa*—a transcript marker for more general intracellular and/or local recognition of virus—to compare responses between Atlantic salmon genotypes and for different PRV-1 genotype exposures. We identified that during PRV-1 infection, *cd8a* transcription was highly correlated (r = 0.86; *p* < 0.001) with *ifng* cytokine transcription (Figure 4A), indirectly indicating that CD8+ T cells were likely becoming locally attracted/activated and generating gamma interferon in response to PRV-1 recognition. Expression of *cd8a* appeared to further be correlated, although to a lesser degree, with general cellular antiviral responsiveness within heart tissues, as measured by *ifna* transcription (r = 0.41; *p* < 0.001) (Figure 4A).

In considering CD8+ T cell involvement in PRV-1-associated heart inflammation, *cd8a* transcription—and by implication CD8+ T cell involvement—was highly associated with heart inflammation in this study. Mild, moderate, or severe heart inflammation was associated with a mean 6- to 17-fold higher *cd8a* transcription relative to fish with no heart inflammation (Figure 4B). In contrast, general intracellular virus recognition as measured by *ifna* transcription was similar in fish with or without heart inflammation (1.1- to 1.4-fold mean increase; Figure 4B). Furthermore, temporal assessments of *cd8a* transcription categorized by either PRV-1 isolate or Atlantic salmon strain indicated that both PRV-1 isolate and Atlantic salmon strain influenced the quantity of heart *cd8a* transcription in response to PRV-1 (Figure 4C,D). Cumulatively, these data support previous findings that recognition of PRV-1-infected cells by CD8+ T cells is likely the main driver of PRV-1-associated heart inflammation in Atlantic salmon and that HSMI is associated with an adaptive immune response. We further identify that PRV-1 activation of CD8+ T cells likely initiates interferon gamma signaling cascades, and is affected to some degree by both PRV-1 and host genotype, whereby BC-PRV was associated with lower CD8+ T cell responses relative to NB- or NOR-PRV, while EU salmon showed higher CD8+ T cell sensitivity than either BC or NB-TR Atlantic salmon during the peak to early persistent phases of infection (6–10 wpc).

### 3.5. Challenge 2: PRV-1-Associated Heart Inflammation Can Be Affected More by Atlantic Salmon Strain than PRV-1 Isolate, Whereas Skeletal Muscle Inflammation Is Influenced Similarly by Both Salmon Strain and PRV-1 Isolate

**Figure 5:**
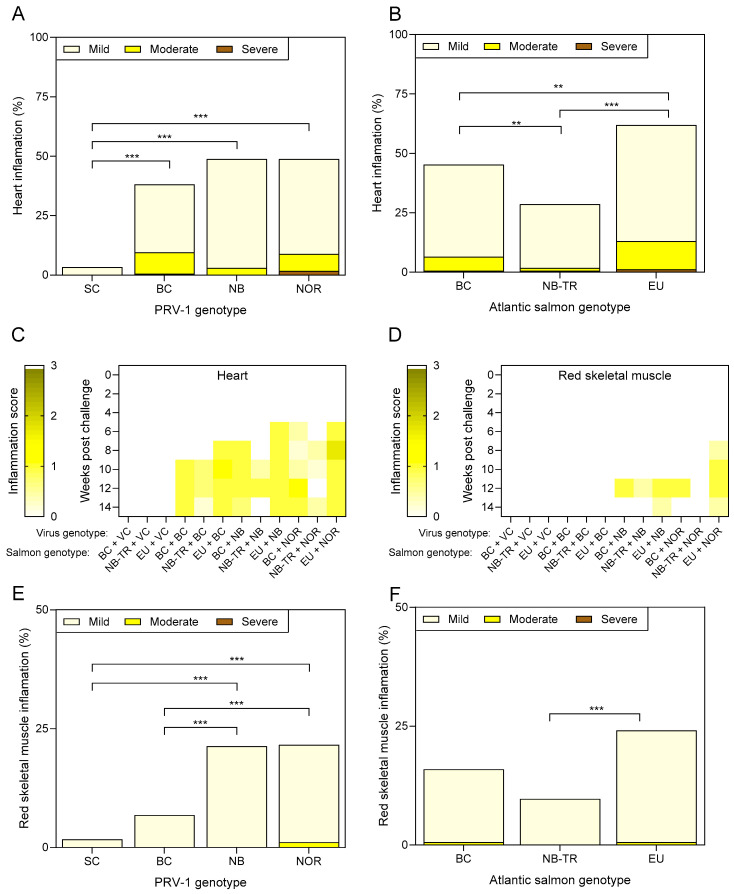
Heart and red skeletal muscle inflammation following PRV-1 challenge. The cumulative prevalence (%) of heart inflammation in PRV-1-challenged salmon over 14 weeks categorized by either (**A**) the PRV-1 isolate administered or (**B**) the strain of the recipient Atlantic salmon; ** *p*  <  0.01, *** *p*  <  0.001 by Kruskal–Wallis and Dunn’s multiple comparisons tests. Heat maps of (**C**) heart and (**D**) red skeletal muscle presented as median inflammatory heart score in relation to each Atlantic salmon x PRV combination over a 14-week progression. Median inflammation score is defined by color, which ranges from 0 (no inflammation; white) to 3 (severe inflammation; brown). The cumulative prevalence (%) of red skeletal muscle inflammation in PRV-1-challenged salmon over the same 14 weeks categorized by either (**E**) the PRV-1 isolate administered or (**F**) the strain of recipient Atlantic salmon is also provided; *** *p*  <  0.001 by Kruskal–Wallis and Dunn’s multiple comparisons tests.

Regarding heart inflammation, the three PRV-1 isolates had similar cumulative impacts on heart inflammation across the three strains of Atlantic salmon used in this study, with approximately 40–50% of sampled fish showing some degree of heart inflammation within the 14-week study for all three PRV-1 isolates administered relative to approximately 3% in SC fish (Figure 5A). However, the impact of Atlantic salmon strain on heart inflammation indicated that NB-TR salmon had the lowest prevalence of heart inflammation (29%, *p* < 0.002), while BC salmon had an intermediate prevalence (45%, *p* < 0.002) and EU salmon had the most heart inflammation (62%, *p* < 0.002) following PRV-1 challenge (Figure 5B). NB-TR salmon also appeared to be the only salmon genotype to show some evidence of heart inflammation resolution within this 14-week study period (Figure 5C).

Skeletal muscle inflammation has been less directly attributable to PRV-1 infection relative to heart inflammation [29], particularly in laboratory challenge trials [1]. Nevertheless, increased red (but not white) skeletal muscle inflammation reached 21% across the three salmon strains following both NB- and NOR-PRV challenge, which was higher than for either the SC or BC-PRV challenge groups (2% and 7% prevalence, respectively; *p* < 0.001; Figure 5E). Median non-zero red skeletal muscle pathology scores at each time point were also only observed in NB- and NOR-PRV-challenged fish (n = 8 per treatment per time point) which occurred between 8–14 wpc (Figure 5D). Salmon strain also appeared to have some influence over red skeletal muscle inflammation in this study, where EU salmon showed a higher prevalence (24%) compared to NB-TR salmon (10%) (Figure 5F).

## 4. Discussion

In two laboratory challenge trials with strict environmental control, we identified that both Atlantic salmon host genetics and PRV-1 genotype influenced disease severity; however, our hypothesis for viral genotype being the main driver of heart inflammation was proved incorrect in this instance—variations in host Atlantic salmon genotype had a stronger influence on the occurrence and severity of heart inflammation than the PRV-1 genotype in these experimental investigations. Similar observations of a strong host phenotypic influence on disease outcomes have also been observed in other reovirus infections of teleost fish (e.g., during Grass Carp reovirus infections) [38].

The distinct phylogenetic ordinations for the three PRV-1 isolates used in this study with high bootstrap support are corroborated by previous Bayesian analyses conducted using virtually the same data set [36] as well as more broadly in PRV-1 phylogenetic investigations [13,39,40]. These data cumulatively lend strong support for a clear phylogenetic distinction between all Northern Pacific PRV-1a isolates sequenced to date including the BC-PRV isolate from PRV-1a isolates obtained or originating from the Atlantic Ocean or coastal Chile, including NB-PRV [40]. These data further support the well-established distinctions between PRV-1a and PRV-1b genotypes, as determined by S1 and whole genome sequencing [31], for which NOR-PRV is clearly classified as PRV-1b and BC-PRV and NB-PRV is clearly classified as PRV-1a.

Given the robust support for separate phylogenetic classifications of the three PRV-1 isolates in this study, it can be presumed that these genetic differences would be mirrored at least to some degree by phenotypic variations as well. We confirm this to be the case—both PRV-1 infection dynamics as well as virulence were at least partially dependent on the genotype of the PRV-1 isolate. Specific to Challenge 2 trials, BC-PRV had the highest capacity for replication, dissemination, and persistence across the three Atlantic salmon strains, while NOR-PRV had the lowest capacity. In a previous study comparing BC-PRV and NOR-PRV infection dynamics within a single strain of European Atlantic salmon [12], it was similarly observed that BC-PRV (i.e., CAN 16-005ND) reached higher systemic blood loads than NOR-PRV (i.e., NOR-2018NL). However, in that study, PRV-1 RNA reached equal if not slightly higher quantities in both the plasma and heart of NOR-PRV-infected fish relative to BC-PRV fish which was the opposite to what was observed in our current study. This suggests there are likely additional factors that influence PRV-1 infection dynamics beyond PRV-1 genetics, which requires further exploration.

In our present study, NOR-PRV demonstrated higher virulence for inducing heart and/or skeletal muscle inflammation relative to BC-PRV, which was consistent with previous challenges comparing these two genotypes [12]. In both previous challenges as well as the challenges conducted here, heart inflammation persisted longer, and median (or mean) heart inflammation scores reached the highest levels in NOR-PRV-infected salmon, although the present study identified this to be confounded to some degree by host genetic factors. Nevertheless, the fact that BC-PRV was able to reach 4–5x higher systemic loads relative to NOR-PRV and still be associated with less cytotoxic T cell responsiveness and heart inflammation in our study strongly supports the supposition that BC-PRV is less virulent than NOR-PRV, and that NB-PRV preliminarily appears to be somewhere in between. We also identified, for the first time, a differential association for generating skeletal muscle inflammation between PRV-1 isolates where both NOR-PRV and NB-PRV were associated with increased red skeletal muscle inflammation (20–25% prevalence), whereas BC-PRV did not notably induce skeletal muscle inflammation beyond control levels (e.g., <8% prevalence). This observation appears to be at least partially supported by field observations in British Columbia vs. Norway, where in BC, skeletal muscle inflammation has been rarely associated with PRV infection [2,8,10,41] whereas in Norway skeletal muscle inflammation has been considerably more common (i.e., common enough to be included in the disease name) [5,6,42]. This may be an important factor to consider in assigning PRV-1 disease pathology in different regions with distinct PRV-1 genotype occurrences.

Cytotoxic T cells have been shown to be highly associated with PRV-1b heart inflammation during HSMI [43], which has also been observed in PRV-1a with milder heart inflammation [23], implying that disease and inflammation associated with PRV-1 infections are generally a result of cytotoxic T cell signaling and directed killing rather than viral egress or pattern recognition receptor activation by infected cells [3]. We confirm that cytotoxic T cells appear to be highly associated with both PRV-1a and -1b heart inflammation in this study, as evidenced by increased *cd8a*, *gzma*, and *ifng* transcriptional signaling, and that this recognition is generally independent from systemic intracellular recognition of the virus by infected cells, as evidenced by a mild systemic and heart-specific *infa* transcriptional response. Thus, the main virulence factors for PRV-1 in Atlantic salmon appear to be how robustly host cytotoxic T cells recognize and respond to the virus in local tissues and not the degree to which tissue or blood cells intracellularly recognize infection. Further, variations in host salmon genotype appeared to be a primary driver of adaptive cytotoxic T cell recognition, where EU salmon mounted a more robust adaptive response to PRV-1 exposure than either BC or NB-TR salmon, indicating that something in the adaptive response of EU fish resulted in better virus recognition, or that viral dissemination into heart tissues and cells was more prevalent in this salmon strain.

Although we saw evidence in this study that PRV-1 genotype can influence cytotoxic T cell recognition and response, variations in viral genetics were clearly outweighed in this instance by genotypic variation in host Atlantic salmon strain. EU Atlantic salmon clearly generated more heart inflammation in response to PRV-1 than either BC or NB salmon, and NB salmon showed the lowest inflammatory responsiveness to PRV-1. These findings appeared to be at least partially consistent across both Challenge 1 and Challenge 2 experiments—BC salmon showed more heart inflammation relative to either of the closely related New Brunswick Atlantic salmon stocks used in this study (NB-SJR in Challenge 1 and NB-TR in Challenge 2). This finding is also consistent with multiple previous challenge trials using BC salmon and BC-PRV that generated less heart inflammation compared to similarly conducted trials that used NOR-PRV in domesticated EU salmon [1,2,11,12,23]. Assuming that this large host effect on determining PRV-1-associated heart inflammation and HSMI can be confirmed through further investigations, host genetics will be important to consider in designing future studies exploring PRV-1 virulence. Specifically, genetic assessments have the potential to identify markers in salmon for establishing resistance to HSMI, thereby reducing production-associated losses in areas where HSMI is problematic. Genome-wide association studies (GWAS) comparing genetic differences between salmon phenotypes for PRV-1 resistance may also be useful in evaluating risks associated with PRV-1 exposure. GWAS studies have shown promise for clarifying the host genetic role and defining risk factors for rheumatic heart disease occurrence in humans [44] with similar epidemiological patterns, which may prove advantageous in Atlantic salmon where GWAS are already relatively common within cultured populations and PRV-1 prevalence is high. Epigenetic factors, particularly relating to past exposure to pathogens, may also prove interesting avenues for investigation for explaining differential responses to PRV-1 by different salmon strains [45,46]. Interestingly, the body of disease prevention work relating to PRV-1—including this present study—indicates that GWAS and other strategies have promise in reducing HSMI-like disease but will likely have little effect on systemic PRV-1 infection dynamics (reviewed by [3]).

Environmental factors are also implicitly important for disease to occur [24]. Although the environment was tightly controlled in this study to allow us to focus on host and virus factors, we nevertheless observed that heart inflammation generated by BC-PRV in BC salmon in the two challenge trials of this study conducted at the Onda Prince Edward Island facility appeared anecdotally more severe when compared to previous challenge trials using the same or similar BC-PRV and BC salmon conducted at a Fisheries and Oceans Canada facility on Vancouver Island [2,11,23]. This would indicate that there are indeed potential differences in environment, even under controlled laboratory conditions, that can impact PRV-1 virulence and which warrant further exploration. Investigations to untangle how environmental impacts might or might not be synergistically tied to host or viral factors may also help to clarify the generally discrete field observations for minimal PRV-1-associated disease in Pacific and Atlantic Canada relative to coastal Norway.

In conclusion, this study identifies that host genetics can exert a majority influence on PRV-1 virulence and associated heart inflammation in controlled laboratory environments, demonstrating that the presence of either PRV-1a or PRV-1b alone is not sufficient to reliably predict disease. This finding also highlights host genotyping and putative genetic selection of Atlantic salmon may prove effective avenues for reducing HSMI disease severity in areas where it is problematic. This study also confirms variation in virulence potential and viral replication between PRV-1 genotypes, particularly regarding replication and an association with generating skeletal muscle inflammation, which may be important to consider in assessing PRV-associated disease or disease risk across geographic landscapes. Lastly, this study reiterates the importance of a host-generated cytotoxic T cell response as a major source of PRV-1-associated disease when it is observed, and, therefore, further research aimed at minimizing and better understanding this response will likely prove useful in mitigating disease in the areas or populations which appear to be at higher risk.

## Figures and Tables

**Table 1:** Matrix of viral inoculates and fish stocks used in the two disease challenge trials presented in this study. PRV-1 isolates—BC-16-005ND (BC-PRV), NOR-2018NL (NOR-PRV), and NB-2018-128 (NB-PRV)—along with saline control inoculate (SC) are indicated, as well as the four fish stocks used: BC—Mowi-McConnell (BC salmon), New Brunswick St. John River (NB-SJR salmon), New Brunswick Tobique River (NB-TR salmon), and European Mowi from Scotland (EU salmon). Mean fish weights obtained from opportunistic subsampling at the start of the challenge are provided.

Challenge Trial	Viral Inoculate	Fish Stock	Mean Fish Weight (g)
1	BC-PRV (purified particles)	BC salmon	36
	SC	BC salmon	42
	BC-PRV (purified particles)	NB-SJR salmon	42
	SC	NB-SJR salmon	44
2	BC-PRV (purified particles)	BC salmon	59
	NB-PRV (blood homogenate)	BC salmon	61
	NOR-PRV (purified particles)	BC salmon	58
	SC	BC salmon	55
	BC-PRV (purified particles)	NB-TR salmon	43
	NB-PRV(blood homogenate)	NB-TR salmon	44
	NOR-PRV (purified particles)	NB-TR salmon	44
	SC	NB-TR salmon	48
	BC-PRV (purified particles)	EU salmon	67
	NB-PRV (blood homogenate)	EU salmon	74
	NOR-PRV (purified particles)	EU salmon	70
	SC	EU salmon	71

## Data Availability

All data presented in this study are available in the manuscript, supplemental material, or freely accessible via NCBI.

## Supplementary Materials

The following supporting information can be downloaded at: https://www.mdpi.com/article/10.3390/v17020285/s1, Figure S1: Histopathology scoring report provided by M.A. and Fish Vet group including 10 images exemplifying inflammatory scoring. Figure S2: Challenge 2 14-week PRV-1 transcriptional blood load for each specific PRV-1 isolate x Atlantic salmon strain challenge; Table S1: Challenge 1 collected and generated data; Table S2: Challenge 2 collected and generated data; Table S3: Tiling primers used for PRV-1 sequencing; Table S4: Atlantic salmon functional sequence comparisons; Table S5: GenBank accession numbers for PRV-1 isolates used in this study.
