# Supplementary material for: PRV-1 Virulence in Atlantic Salmon Is Affected by Host Genotype
Source: Viruses. 2025 Feb 19;17(2):285. doi: 10.3390/v17020285 (PMC11860446; doi:10.3390/v17020285)
Supplement: Supplementary file 1 [file viruses-17-00285-s001.zip › viruses-3471247-supplementary/viruses-3471247-SM/viruses-3471247-Figure S1.pdf]

FishVet Group

Report: Histology

REPORT NO.
FVG-YZ53EKTB-RH

ORDERED AS
Project (6 weeks)

CASE PATHOLOGIST
Alarcón, Marta

REPORTED ON
23.12.2020

Customer

CLIENT
Fisheries and Oceans Canada

SUBMITTED BY
Garver, Kyle
(Fisheries and Oceans Canada)

REPORT TO
Garver, Kyle (Fisheries and Oceans Canada)
Garver, Kyle (Fisheries and Oceans

INVOICE TO
Fisheries and Oceans Canada

SUBMITTERS REFERENCE NO.
PRV-1 multi-strain comparison

Details of Sample Materials

SITE NAME
Pacific Biological Station

SAMPLE DATE

RECEIVED DATE
09.10.2020

MS SITE REF
CA-0001

STOCK TYPE

TRANSFER DATE

SAMPLE NUMBER
1-144

SAMPLE TYPE
Formalin fixed tissue

SPECIES
Atlantic salmon

COMMENTS

Findings

Assessing the severity of inflammation in 155 organs (144 hearts + 10 skeletal muscle) from a PRV challenge experiment.

The scoring was performed blindly as in the following paper: Wessel Ø, Braaen S, Alarcon M, Haatveit H, Roos N, Markussen T, et al. (2017) Infection with purified Piscine orthoreovirus demonstrates a causal relationship with heart and skeletal muscle inflammation in Atlantic salmon. PLoS ONE 12(8): e0183781. https://doi.org/10.1371/journal.pone.0183781

Conclusions/Comments

Details of the score are provided in the excel file

Marta Alarcón
veterinær

Additional Information

TRANSFER DATE	AVERAGE WEIGHT	WATER TYPE	WATER TEMPERATURE	VACCINE	SUSPICION/ASSUMPTION
	30 g	Seawater	10 °C		

Obtained samples - detailed list

INDIVIDUAL	SAMPLE NO.	SAMPLE TYPE	ID	BARCODE	CAGE	BATCH	TISSUES
QCATC259 - Week 6 - D1-F7 - Fish 2	1	Formalin fixed tissue	QCATC259 - Week 6 - D1-F7 - Fish 2	FVG-D5TPWZ36			Heart
QCATC259 - Week 6 - D1-F7 - Fish 3	2	Formalin fixed tissue	QCATC259 - Week 6 - D1-F7 - Fish 3	FVG-PVGP2HFQ			Heart
QCATC259 - Week 6 - D1-F7 - Fish 4	3	Formalin fixed tissue	QCATC259 - Week 6 - D1-F7 - Fish 4	FVG-PNNPBV94			Heart
QCATC259 - Week 6 - D2-F8 - Fish 1	4	Formalin fixed tissue	QCATC259 - Week 6 - D2-F8 - Fish 1	FVG-JKVG96TW			Heart
QCATC259 - Week 6 - D2-F8 - Fish 2	5	Formalin fixed tissue	QCATC259 - Week 6 - D2-F8 - Fish 2	FVG-EGBCV7A7			Heart
QCATC259 - Week 6 - E1-F9 - Fish 2	6	Formalin fixed tissue	QCATC259 - Week 6 - E1-F9 - Fish 2	FVG-ME29E7E2			Heart
QCATC259 - Week 6 - E1-F9 - Fish 3	7	Formalin fixed tissue	QCATC259 - Week 6 - E1-F9 - Fish 3	FVG-HTZC6QE6			Heart
QCATC259 - Week 6 - E1-F9 - Fish 4	8	Formalin fixed tissue	QCATC259 - Week 6 - E1-F9 - Fish 4	FVG-TDYFS2E7			Heart
QCATC259 - Week 6 - E2-F10 - Fish 1	9	Formalin fixed tissue	QCATC259 - Week 6 - E2-F10 - Fish 1	FVG-JVG54FV5			Heart
QCATC259 - Week 6 - E2-F10 - Fish 2	10	Formalin fixed tissue	QCATC259 - Week 6 - E2-F10 - Fish 2	FVG-D4V2V346			Heart
QCATC259 - Week 6 - F1-F11 - Fish 2	11	Formalin fixed tissue	QCATC259 - Week 6 - F1-F11 - Fish 2	FVG-3572ZXEK			Heart
QCATC259 - Week 6 - F1-F11 - Fish 3	12	Formalin fixed tissue	QCATC259 - Week 6 - F1-F11 - Fish 3	FVG-MGQ9X8UZ			Heart
QCATC259 - Week 6 - F1-F11 - Fish 4	13	Formalin fixed tissue	QCATC259 - Week 6 - F1-F11 - Fish 4	FVG-5SR594P3			Heart
QCATC259 - Week 6 - F2-F12 - Fish 1	14	Formalin fixed tissue	QCATC259 - Week 6 - F2-F12 - Fish 1	FVG-D8874PEX			Heart
QCATC259 - Week 6 - F2-F12 - Fish 2	15	Formalin fixed tissue	QCATC259 - Week 6 - F2-F12 - Fish 2	FVG-TGG7VYMC			Heart
QCATC259 - Week 6 - G1-F13 - Fish 2	16	Formalin fixed tissue	QCATC259 - Week 6 - G1-F13 - Fish 2	FVG-8T4ENFW6			Heart
QCATC259 - Week 6 - G1-F13 - Fish 3	17	Formalin fixed tissue	QCATC259 - Week 6 - G1-F13 - Fish 3	FVG-FFTWS7QR			Heart
QCATC259 - Week 6 - G1-F13 - Fish 4	18	Formalin fixed tissue	QCATC259 - Week 6 - G1-F13 - Fish 4	FVG-RW7BUG7N			Heart
QCATC259 - Week 6 - G2-F14 - Fish 1	19	Formalin fixed tissue	QCATC259 - Week 6 - G2-F14 - Fish 1	FVG-KN3AH9HX			Heart
QCATC259 - Week 6 - G2-F14 - Fish 2	20	Formalin fixed tissue	QCATC259 - Week 6 - G2-F14 - Fish 2	FVG-SKPURZWC			Heart
QCATC259 - Week 6 - H1-F15 - Fish 3	21	Formalin fixed tissue	QCATC259 - Week 6 - H1-F15 - Fish 3	FVG-8H9PU6X8			Heart
QCATC259 - Week 6 - H1-F15 - Fish 4	22	Formalin fixed tissue	QCATC259 - Week 6 - H1-F15 - Fish 4	FVG-HCJC8WDP			Heart
QCATC259 - Week 6 - H2-F16 - Fish 1	23	Formalin fixed tissue	QCATC259 - Week 6 - H2-F16 - Fish 1	FVG-B2ZBG6EN			Heart

INDIVIDUAL	SAMPLE NO.	SAMPLE TYPE	ID	BARCODE	CAGE	BATCH	TISSUES
QCATC259 - Week 6 - H2-F16 - Fish 2	24	Formalin fixed tissue	QCATC259 - Week 6 - H2-F16 - Fish 2	FVG-88GBZT6B			Heart
QCATC259 - Week 6 - H2-F16 - Fish 3	25	Formalin fixed tissue	QCATC259 - Week 6 - H2-F16 - Fish 3	FVG-84SSE3RA			Heart
QCATC259 - Week 6 - I1-F17 - Fish 2	26	Formalin fixed tissue	QCATC259 - Week 6 - I1-F17 - Fish 2	FVG-832DX7RB			Heart
QCATC259 - Week 6 - I1-F17 - Fish 3	27	Formalin fixed tissue	QCATC259 - Week 6 - I1-F17 - Fish 3	FVG-2BAFD479			Heart
QCATC259 - Week 6 - I1-F17 - Fish 4	28	Formalin fixed tissue	QCATC259 - Week 6 - I1-F17 - Fish 4	FVG-VB7CD6A7			Heart
QCATC259 - Week 6 - I2-F18 - Fish 1	29	Formalin fixed tissue	QCATC259 - Week 6 - I2-F18 - Fish 1	FVG-XEGB27MA			Heart
QCATC259 - Week 6 - I2-F18 - Fish 2	30	Formalin fixed tissue	QCATC259 - Week 6 - I2-F18 - Fish 2	FVG-XGB7E4BU			Heart
QCATC259 - Week 6 - J1-F19 - Fish 3	31	Formalin fixed tissue	QCATC259 - Week 6 - J1-F19 - Fish 3	FVG-GMG5ZV3V			Heart
QCATC259 - Week 6 - J1-F19 - Fish 4	32	Formalin fixed tissue	QCATC259 - Week 6 - J1-F19 - Fish 4	FVG-2U9TXVX6			Heart
QCATC259 - Week 6 - J2-F20 - Fish 1	33	Formalin fixed tissue	QCATC259 - Week 6 - J2-F20 - Fish 1	FVG-SNNUB2B7			Heart
QCATC259 - Week 6 - J2-F20 - Fish 2	34	Formalin fixed tissue	QCATC259 - Week 6 - J2-F20 - Fish 2	FVG-ZZ8839E6			Heart
QCATC259 - Week 6 - J2-F20 - Fish 3	35	Formalin fixed tissue	QCATC259 - Week 6 - J2-F20 - Fish 3	FVG-K3WP2CV5			Heart
QCATC259 - Week 6 - K1-F21 - Fish 3	36	Formalin fixed tissue	QCATC259 - Week 6 - K1-F21 - Fish 3	FVG-E6NBFN5V			Heart
QCATC259 - Week 6 - K1-F21 - Fish 4	37	Formalin fixed tissue	QCATC259 - Week 6 - K1-F21 - Fish 4	FVG-X8KA8MHD			Heart
QCATC259 - Week 6 - K2-F22 - Fish 1	38	Formalin fixed tissue	QCATC259 - Week 6 - K2-F22 - Fish 1	FVG-8TX698HU			Heart
QCATC259 - Week 6 - K2-F22 - Fish 2	39	Formalin fixed tissue	QCATC259 - Week 6 - K2-F22 - Fish 2	FVG-DFPET2C2			Heart
QCATC259 - Week 6 - K2-F22 - Fish 3	40	Formalin fixed tissue	QCATC259 - Week 6 - K2-F22 - Fish 3	FVG-Y2H7FSM2			Heart
QCATC259 - Week 6 - L1-F23 - Fish 3	41	Formalin fixed tissue	QCATC259 - Week 6 - L1-F23 - Fish 3	FVG-8U8QAYT3			Heart
QCATC259 - Week 6 - L1-F23 - Fish 4	42	Formalin fixed tissue	QCATC259 - Week 6 - L1-F23 - Fish 4	FVG-QP6AFH9N			Heart
QCATC259 - Week 6 - L2-F24 - Fish 1	43	Formalin fixed tissue	QCATC259 - Week 6 - L2-F24 - Fish 1	FVG-69PEW2H8			Heart
QCATC259 - Week 6 - L2-F24 - Fish 2	44	Formalin fixed tissue	QCATC259 - Week 6 - L2-F24 - Fish 2	FVG-8FXKWY5A			Heart
QCATC259 - Week 6 - L2-F24 - Fish 3	45	Formalin fixed tissue	QCATC259 - Week 6 - L2-F24 - Fish 3	FVG-S38BR3TP			Heart
QCATC259 - Week 8 - D1-F7 - Fish 3	46	Formalin fixed tissue	QCATC259 - Week 8 - D1-F7 - Fish 3	FVG-4WS7BHMK			Heart
QCATC259 - Week 8 - D1-F7 - Fish 4	47	Formalin fixed tissue	QCATC259 - Week 8 - D1-F7 - Fish 4	FVG-AWZC48T8			Heart
QCATC259 - Week 8 - D2-F8 - Fish 1	48	Formalin fixed tissue	QCATC259 - Week 8 - D2-F8 - Fish 1	FVG-G729YMV8			Heart
QCATC259 - Week 8 - D2-F8 - Fish 2	49	Formalin fixed tissue	QCATC259 - Week 8 - D2-F8 - Fish 2	FVG-R8UTKEH5			Heart

INDIVIDUAL	SAMPLE NO.	SAMPLE TYPE	ID	BARCODE	CAGE	BATCH	TISSUES
QCATC259 - Week 8 - D2-F8 - Fish 3	50	Formalin fixed tissue	QCATC259 - Week 8 - D2-F8 - Fish 3	FVG-UNBCC7MY			Heart
QCATC259 - Week 8 - E1-F9 - Fish 3	51	Formalin fixed tissue	QCATC259 - Week 8 - E1-F9 - Fish 3	FVG-DGR9GRNP			Heart
QCATC259 - Week 8 - E1-F9 - Fish 4	52	Formalin fixed tissue	QCATC259 - Week 8 - E1-F9 - Fish 4	FVG-9949X9X4			Heart
QCATC259 - Week 8 - E2-F10 - Fish 1	53	Formalin fixed tissue	QCATC259 - Week 8 - E2-F10 - Fish 1	FVG-7F5CVSZ8			Heart
QCATC259 - Week 8 - E2-F10 - Fish 2	54	Formalin fixed tissue	QCATC259 - Week 8 - E2-F10 - Fish 2	FVG-S7FZ53YF			Heart
QCATC259 - Week 8 - E2-F10 - Fish 3	55	Formalin fixed tissue	QCATC259 - Week 8 - E2-F10 - Fish 3	FVG-UGZ74WQ4			Heart
QCATC259 - Week 8 - F1-F11 - Fish 3	56	Formalin fixed tissue	QCATC259 - Week 8 - F1-F11 - Fish 3	FVG-37HDP3JS			Heart
QCATC259 - Week 8 - F1-F11 - Fish 4	57	Formalin fixed tissue	QCATC259 - Week 8 - F1-F11 - Fish 4	FVG-P4RDCJ2B			Heart
QCATC259 - Week 8 - F2-F12 - Fish 1	58	Formalin fixed tissue	QCATC259 - Week 8 - F2-F12 - Fish 1	FVG-D9E73Y4Z			Heart
QCATC259 - Week 8 - F2-F12 - Fish 2	59	Formalin fixed tissue	QCATC259 - Week 8 - F2-F12 - Fish 2	FVG-Z9BFUH53			Heart
QCATC259 - Week 8 - F2-F12 - Fish 3	60	Formalin fixed tissue	QCATC259 - Week 8 - F2-F12 - Fish 3	FVG-9NK4EGTU			Heart
QCATC259 - Week 8 - G1-F13 - Fish 3	61	Formalin fixed tissue	QCATC259 - Week 8 - G1-F13 - Fish 3	FVG-C6GD9PGF			Heart
QCATC259 - Week 8 - G1-F13 - Fish 4	62	Formalin fixed tissue	QCATC259 - Week 8 - G1-F13 - Fish 4	FVG-39U6EG9R			Heart
QCATC259 - Week 8 - G2-F14 - Fish 1	63	Formalin fixed tissue	QCATC259 - Week 8 - G2-F14 - Fish 1	FVG-276B3VUW			Heart
QCATC259 - Week 8 - G2-F14 - Fish 2	64	Formalin fixed tissue	QCATC259 - Week 8 - G2-F14 - Fish 2	FVG-SP97K9V2			Heart
QCATC259 - Week 8 - G2-F14 - Fish 3	65	Formalin fixed tissue	QCATC259 - Week 8 - G2-F14 - Fish 3	FVG-4JTKPE38			Heart
QCATC259 - Week 8 - H1-F15 - Fish 3	66	Formalin fixed tissue	QCATC259 - Week 8 - H1-F15 - Fish 3	FVG-B58T4YGT			Heart
QCATC259 - Week 8 - H1-F15 - Fish 4	67	Formalin fixed tissue	QCATC259 - Week 8 - H1-F15 - Fish 4	FVG-VP9N8462			Heart
QCATC259 - Week 8 - H2-F16 - Fish 1	68	Formalin fixed tissue	QCATC259 - Week 8 - H2-F16 - Fish 1	FVG-45HQQJ66			Heart
QCATC259 - Week 8 - H2-F16 - Fish 2	69	Formalin fixed tissue	QCATC259 - Week 8 - H2-F16 - Fish 2	FVG-Z29E38A5			Heart
QCATC259 - Week 8 - H2-F16 - Fish 3	70	Formalin fixed tissue	QCATC259 - Week 8 - H2-F16 - Fish 3	FVG-9TFYER6C			Heart
QCATC259 - Week 8 - I1-F17 - Fish 2	71	Formalin fixed tissue	QCATC259 - Week 8 - I1-F17 - Fish 2	FVG-Q6PS9YR6			Heart
QCATC259 - Week 8 - I1-F17 - Fish 3	72	Formalin fixed tissue	QCATC259 - Week 8 - I1-F17 - Fish 3	FVG-9Q68H48W			Heart
QCATC259 - Week 8 - I1-F17 - Fish 4	73	Formalin fixed tissue	QCATC259 - Week 8 - I1-F17 - Fish 4	FVG-GE6MGF3E			Heart
QCATC259 - Week 8 - I2-F18 - Fish 1	74	Formalin fixed tissue	QCATC259 - Week 8 - I2-F18 - Fish 1	FVG-YBFZQNFT			Heart
QCATC259 - Week 8 - I2-F18 - Fish 2	75	Formalin fixed tissue	QCATC259 - Week 8 - I2-F18 - Fish 2	FVG-QGFZMWCN			Heart

INDIVIDUAL	SAMPLE NO.	SAMPLE TYPE	ID	BARCODE	CAGE	BATCH	TISSUES
QCATC259 - Week 8 - J1-F19 - Fish 3	76	Formalin fixed tissue	QCATC259 - Week 8 - J1-F19 - Fish 3	FVG-8VRZ2TX2			Heart
QCATC259 - Week 8 - J1-F19 - Fish 4	77	Formalin fixed tissue	QCATC259 - Week 8 - J1-F19 - Fish 4	FVG-JYV9CVEW			Heart
QCATC259 - Week 8 - J2-F20 - Fish 1	78	Formalin fixed tissue	QCATC259 - Week 8 - J2-F20 - Fish 1	FVG-X4EJZ8SP			Heart
QCATC259 - Week 8 - J2-F20 - Fish 2	79	Formalin fixed tissue	QCATC259 - Week 8 - J2-F20 - Fish 2	FVG-UQRU5DQW			Heart
QCATC259 - Week 8 - J2-F20 - Fish 3	80	Formalin fixed tissue	QCATC259 - Week 8 - J2-F20 - Fish 3	FVG-257XCVPX			Heart
QCATC259 - Week 8 - K1-F21 - Fish 3	81	Formalin fixed tissue	QCATC259 - Week 8 - K1-F21 - Fish 3	FVG-VWDNN62W			Heart
QCATC259 - Week 8 - K1-F21 - Fish 4	82	Formalin fixed tissue	QCATC259 - Week 8 - K1-F21 - Fish 4	FVG-SDAE2B7J			Heart
QCATC259 - Week 8 - K2-F22 - Fish 1	83	Formalin fixed tissue	QCATC259 - Week 8 - K2-F22 - Fish 1	FVG-MXB6H3WK			Heart
QCATC259 - Week 8 - K2-F22 - Fish 2	84	Formalin fixed tissue	QCATC259 - Week 8 - K2-F22 - Fish 2	FVG-A9F892WS			Heart
QCATC259 - Week 8 - K2-F22 - Fish 3	85	Formalin fixed tissue	QCATC259 - Week 8 - K2-F22 - Fish 3	FVG-8CTXWWQY			Heart
QCATC259 - Week 8 - L1-F23 - Fish 3	86	Formalin fixed tissue	QCATC259 - Week 8 - L1-F23 - Fish 3	FVG-8X7NSSU2			Heart
QCATC259 - Week 8 - L1-F23 - Fish 4	87	Formalin fixed tissue	QCATC259 - Week 8 - L1-F23 - Fish 4	FVG-ZE847YAS			Heart
QCATC259 - Week 8 - L2-F24 - Fish 1	88	Formalin fixed tissue	QCATC259 - Week 8 - L2-F24 - Fish 1	FVG-3BSTCDP9			Heart
QCATC259 - Week 8 - L2-F24 - Fish 2	89	Formalin fixed tissue	QCATC259 - Week 8 - L2-F24 - Fish 2	FVG-9TU8C59Q			Heart
QCATC259 - Week 8 - L2-F24 - Fish 3	90	Formalin fixed tissue	QCATC259 - Week 8 - L2-F24 - Fish 3	FVG-DYN9J3ND			Heart
QCATC259 - Week 10 - A2-F2 - Fish 4	91	Formalin fixed tissue	QCATC259 - Week 10 - A2-F2 - Fish 4	FVG-5WETBFUN			Heart/Muscle (2x)
QCATC259 - Week 10 - D2- F8 - Fish 1	92	Formalin fixed tissue	QCATC259 - Week 10 - D2- F8 - Fish 1	FVG-JJXAYUZY			Heart/Muscle (2x)
QCATC259 - Week 10 - D2- F8 - Fish 3	93	Formalin fixed tissue	QCATC259 - Week 10 - D2- F8 - Fish 3	FVG-HMWB59TP			Heart/Muscle (2x)
QCATC259 - Week 10 - F1-F11 - Fish 4	94	Formalin fixed tissue	QCATC259 - Week 10 - F1-F11 - Fish 4	FVG-XV3GNZUS			Heart/Muscle (2x)
QCATC259 - Week 10 - F2-F12 - Fish 1	95	Formalin fixed tissue	QCATC259 - Week 10 - F2-F12 - Fish 1	FVG-7ZKEKV42			Heart/Muscle (2x)
QCATC259 - Week 10 - J1-F19 - Fish 2	96	Formalin fixed tissue	QCATC259 - Week 10 - J1-F19 - Fish 2	FVG-ZAMK6X6X			Heart/Muscle (2x)
QCATC259 - Week 10 - K1- F21 - Fish 3	97	Formalin fixed tissue	QCATC259 - Week 10 - K1- F21 - Fish 3	FVG-HM9MPKKX			Heart/Muscle (2x)
QCATC259 - Week 10 - L1-F23 - Fish 4	98	Formalin fixed tissue	QCATC259 - Week 10 - L1-F23 - Fish 4	FVG-JVG4JJA5			Heart/Muscle (2x)
QCATC259 - Week 10 - L2-F24 - Fish 1	99	Formalin fixed tissue	QCATC259 - Week 10 - L2-F24 - Fish 1	FVG-E7V8B3JE			Heart/Muscle (2x)
QCATC259 - Week 12 - D1- F7 - Fish 3	100	Formalin fixed tissue	QCATC259 - Week 12 - D1- F7 - Fish 3	FVG-45E3A57X			Heart

INDIVIDUAL	SAMPLE NO.	SAMPLE TYPE	ID	BARCODE	CAGE	BATCH	TISSUES
QCATC259 - Week 12 - D1- F7 - Fish 4	101	Formalin fixed tissue	QCATC259 - Week 12 - D1- F7 - Fish 4	FVG-422P2HK6			Heart
QCATC259 - Week 12 - D2- F8 - Fish 1	102	Formalin fixed tissue	QCATC259 - Week 12 - D2- F8 - Fish 1	FVG-4ZX868FJ			Heart
QCATC259 - Week 12 - D2- F8 - Fish 2	103	Formalin fixed tissue	QCATC259 - Week 12 - D2- F8 - Fish 2	FVG-Z37SN5U7			Heart
QCATC259 - Week 12 - D2- F8 - Fish 3	104	Formalin fixed tissue	QCATC259 - Week 12 - D2- F8 - Fish 3	FVG-VXTFMWHB			Heart
QCATC259 - Week 12 - E1- F9 - Fish 3	105	Formalin fixed tissue	QCATC259 - Week 12 - E1- F9 - Fish 3	FVG-5VQWY2D6			Heart
QCATC259 - Week 12 - E1- F9 - Fish 4	106	Formalin fixed tissue	QCATC259 - Week 12 - E1- F9 - Fish 4	FVG-FW38PFCE			Heart
QCATC259 - Week 12 - E2- F10 - Fish 1	107	Formalin fixed tissue	QCATC259 - Week 12 - E2- F10 - Fish 1	FVG-F8DSPKH3			Heart/Muscle (2x)
QCATC259 - Week 12 - E2- F10 - Fish 2	108	Formalin fixed tissue	QCATC259 - Week 12 - E2- F10 - Fish 2	FVG-CXSZ3YXC			Heart
QCATC259 - Week 12 - E2- F10 - Fish 3	109	Formalin fixed tissue	QCATC259 - Week 12 - E2- F10 - Fish 3	FVG-8TY9MMZP			Heart
QCATC259 - Week 12 - F1- F11 - Fish 2	110	Formalin fixed tissue	QCATC259 - Week 12 - F1- F11 - Fish 2	FVG-X7QYC2F2			Heart
QCATC259 - Week 12 - F1- F11 - Fish 3	111	Formalin fixed tissue	QCATC259 - Week 12 - F1- F11 - Fish 3	FVG-YG6PJC2J			Heart
QCATC259 - Week 12 - F1- F11 - Fish 4	112	Formalin fixed tissue	QCATC259 - Week 12 - F1- F11 - Fish 4	FVG-9CW92Y4N			Heart
QCATC259 - Week 12 - F2- F12 - Fish 1	113	Formalin fixed tissue	QCATC259 - Week 12 - F2- F12 - Fish 1	FVG-C7632DVU			Heart
QCATC259 - Week 12 - F2- F12 - Fish 2	114	Formalin fixed tissue	QCATC259 - Week 12 - F2- F12 - Fish 2	FVG-Z7DWFPCS			Heart
QCATC259 - Week 12 - G1- F13 - Fish 2	115	Formalin fixed tissue	QCATC259 - Week 12 - G1- F13 - Fish 2	FVG-PJYF5CGC			Heart
QCATC259 - Week 12 - G1- F13 - Fish 3	116	Formalin fixed tissue	QCATC259 - Week 12 - G1- F13 - Fish 3	FVG-9P6JHKJ9			Heart
QCATC259 - Week 12 - G1- F13 - Fish 4	117	Formalin fixed tissue	QCATC259 - Week 12 - G1- F13 - Fish 4	FVG-J8CKH3PK			Heart
QCATC259 - Week 12 - G2- F14 - Fish 1	118	Formalin fixed tissue	QCATC259 - Week 12 - G2- F14 - Fish 1	FVG-47A7ZEPD			Heart
QCATC259 - Week 12 - G2- F14 - Fish 2	119	Formalin fixed tissue	QCATC259 - Week 12 - G2- F14 - Fish 2	FVG-4TQNBHYR			Heart
QCATC259 - Week 12 - H1- F15 - Fish 3	120	Formalin fixed tissue	QCATC259 - Week 12 - H1- F15 - Fish 3	FVG-SD4B9GDJ			Heart
QCATC259 - Week 12 - H1- F15 - Fish 4	121	Formalin fixed tissue	QCATC259 - Week 12 - H1- F15 - Fish 4	FVG-6D94C39E			Heart

INDIVIDUAL	SAMPLE NO.	SAMPLE TYPE	ID	BARCODE	CAGE	BATCH	TISSUES
QCATC259 - Week 12 - H2- F16 - Fish 1	122	Formalin fixed tissue	QCATC259 - Week 12 - H2- F16 - Fish 1	FVG-TQMDCZX5			Heart
QCATC259 - Week 12 - H2- F16 - Fish 2	123	Formalin fixed tissue	QCATC259 - Week 12 - H2- F16 - Fish 2	FVG-34VJBCBP			Heart
QCATC259 - Week 12 - H2- F16 - Fish 3	124	Formalin fixed tissue	QCATC259 - Week 12 - H2- F16 - Fish 3	FVG-WE4DCD2D			Heart
QCATC259 - Week 12 - I1- F17 - Fish 3	125	Formalin fixed tissue	QCATC259 - Week 12 - I1- F17 - Fish 3	FVG-MZ7CN4YM			Heart
QCATC259 - Week 12 - I1- F17 - Fish 4	126	Formalin fixed tissue	QCATC259 - Week 12 - I1- F17 - Fish 4	FVG-T9XCK92Y			Heart
QCATC259 - Week 12 - I2- F18 - Fish 1	127	Formalin fixed tissue	QCATC259 - Week 12 - I2- F18 - Fish 1	FVG-Z7DNA279			Heart
QCATC259 - Week 12 - I2- F18 - Fish 2	128	Formalin fixed tissue	QCATC259 - Week 12 - I2- F18 - Fish 2	FVG-3QV68Z29			Heart
QCATC259 - Week 12 - I2- F18 - Fish 3	129	Formalin fixed tissue	QCATC259 - Week 12 - I2- F18 - Fish 3	FVG-VX4U6AY3			Heart
QCATC259 - Week 12 - J1- F19 - Fish 2	130	Formalin fixed tissue	QCATC259 - Week 12 - J1- F19 - Fish 2	FVG-FDGGGHK9			Heart
QCATC259 - Week 12 - J1- F19 - Fish 3	131	Formalin fixed tissue	QCATC259 - Week 12 - J1- F19 - Fish 3	FVG-4CCF7EVQ			Heart
QCATC259 - Week 12 - J1- F19 - Fish 4	132	Formalin fixed tissue	QCATC259 - Week 12 - J1- F19 - Fish 4	FVG-4WT4E4D4			Heart/Muscle (2x)
QCATC259 - Week 12 - J2- F20 - Fish 1	133	Formalin fixed tissue	QCATC259 - Week 12 - J2- F20 - Fish 1	FVG-39HK9H2G			Heart
QCATC259 - Week 12 - J2- F20 - Fish 2	134	Formalin fixed tissue	QCATC259 - Week 12 - J2- F20 - Fish 2	FVG-PYKK3NJF			Heart
QCATC259 - Week 12 - K1- F21 - Fish 2	135	Formalin fixed tissue	QCATC259 - Week 12 - K1- F21 - Fish 2	FVG-VZT69KSK			Heart
QCATC259 - Week 12 - K1- F21 - Fish 3	136	Formalin fixed tissue	QCATC259 - Week 12 - K1- F21 - Fish 3	FVG-6N4BHU5W			Heart
QCATC259 - Week 12 - K1- F21 - Fish 4	137	Formalin fixed tissue	QCATC259 - Week 12 - K1- F21 - Fish 4	FVG-J7FURT9H			Heart
QCATC259 - Week 12 - K2- F22 - Fish 1	138	Formalin fixed tissue	QCATC259 - Week 12 - K2- F22 - Fish 1	FVG-Q96XDAVA			Heart
QCATC259 - Week 12 - K2- F22 - Fish 2	139	Formalin fixed tissue	QCATC259 - Week 12 - K2- F22 - Fish 2	FVG-NK7PCWFP			Heart
QCATC259 - Week 12 - L1- F23 - Fish 2	140	Formalin fixed tissue	QCATC259 - Week 12 - L1- F23 - Fish 2	FVG-D8GSB95E			Heart
QCATC259 - Week 12 - L1- F23 - Fish 3	141	Formalin fixed tissue	QCATC259 - Week 12 - L1- F23 - Fish 3	FVG-X58EX4DT			Heart
QCATC259 - Week 12 - L1- F23 - Fish 4	142	Formalin fixed tissue	QCATC259 - Week 12 - L1- F23 - Fish 4	FVG-85JREWXZ			Heart
QCATC259 - Week 12 - L2- F24 - Fish 1	143	Formalin fixed tissue	QCATC259 - Week 12 - L2- F24 - Fish 1	FVG-QR4VMPHF			Heart
QCATC259 - Week 12 - L2- F24 - Fish 2	144	Formalin fixed tissue	QCATC259 - Week 12 - L2- F24 - Fish 2	FVG-DNZQ5E2W			Heart

Images

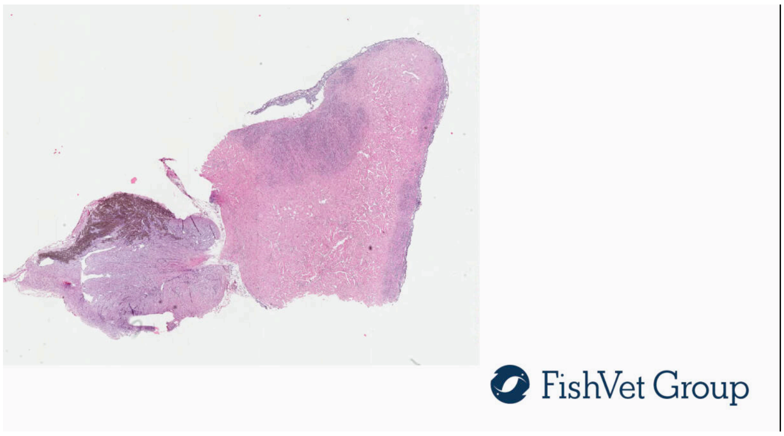

Sample 45. Heart overview. Score 2.3

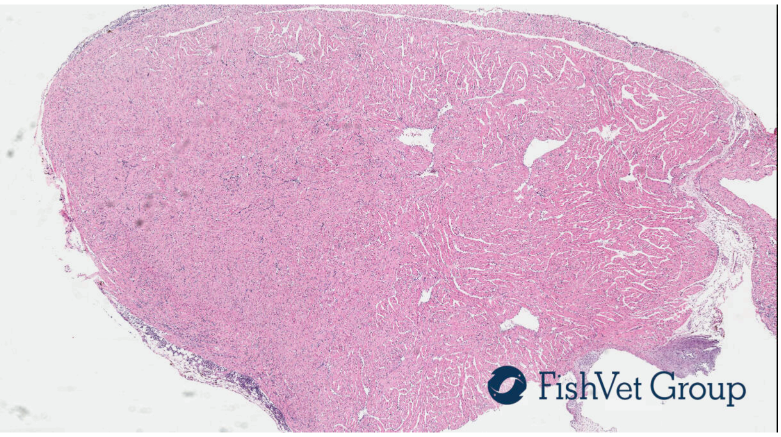

Sample 50. Heart overview. Score 1.3

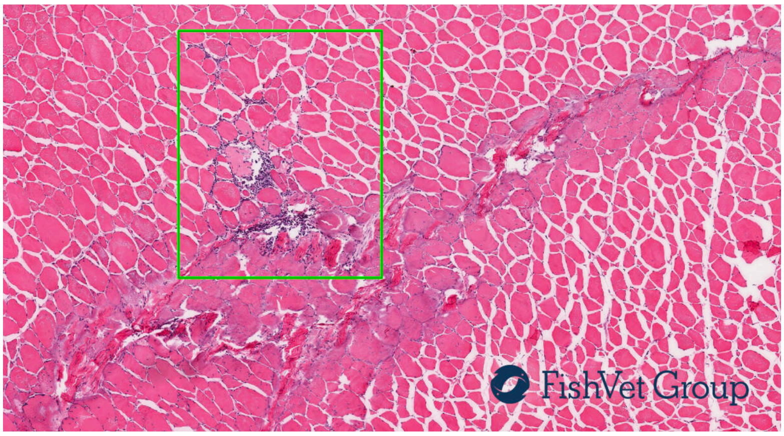

Sample 87. White skeletal muscle. Focal inflammation

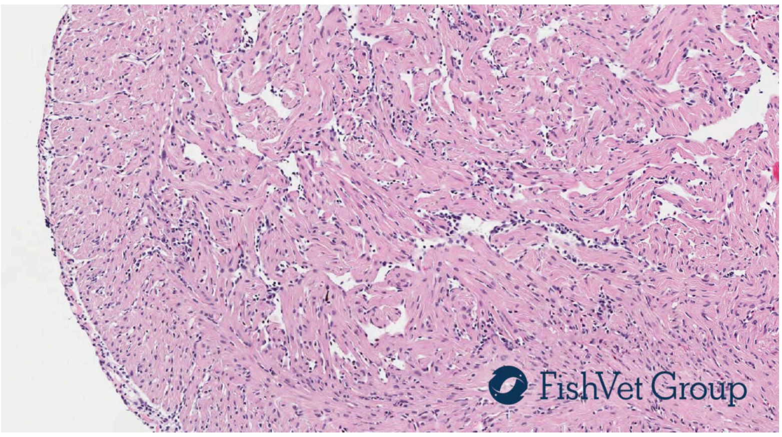

Sample 92. Heart. Spongiosum 1.5

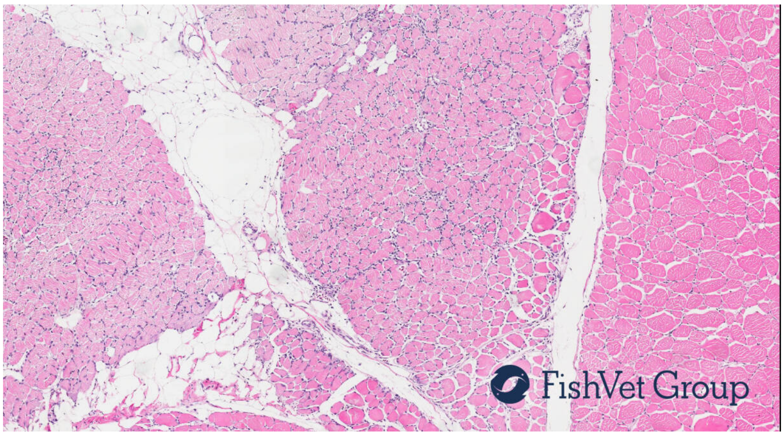

Sample 98. Red skeletal muscle. Score 1

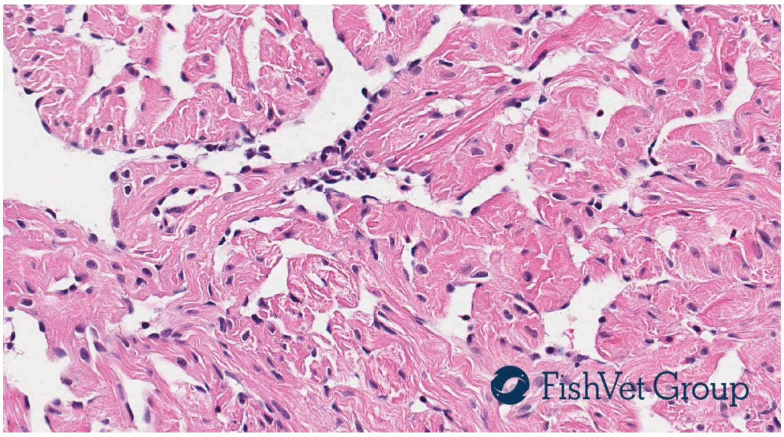

Sample 104. Heart. Spongiosum. Score 0.1

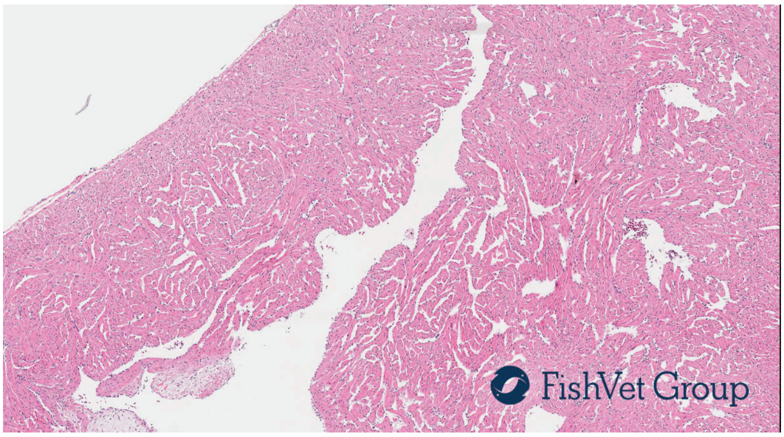

Sample 106. Heart. No lesions observed

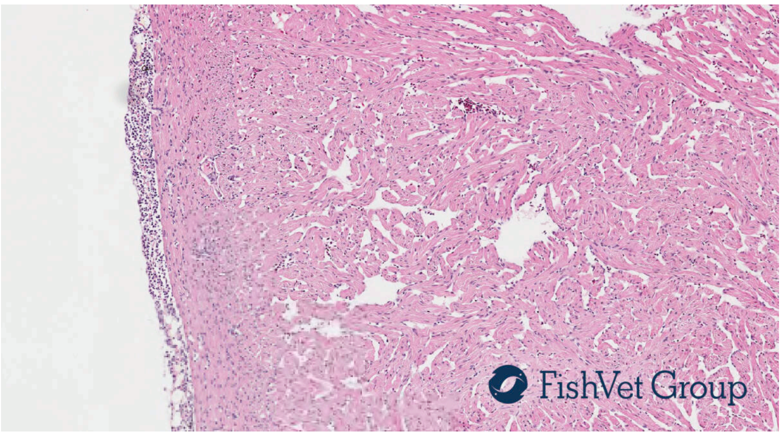

Sample 112. Heart. Epicardium Score 1

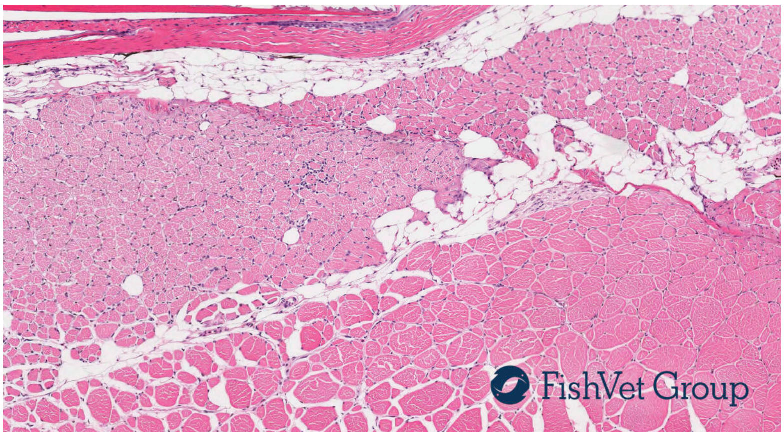

Sample 116. Red skeletal muscle. Focal inflammation

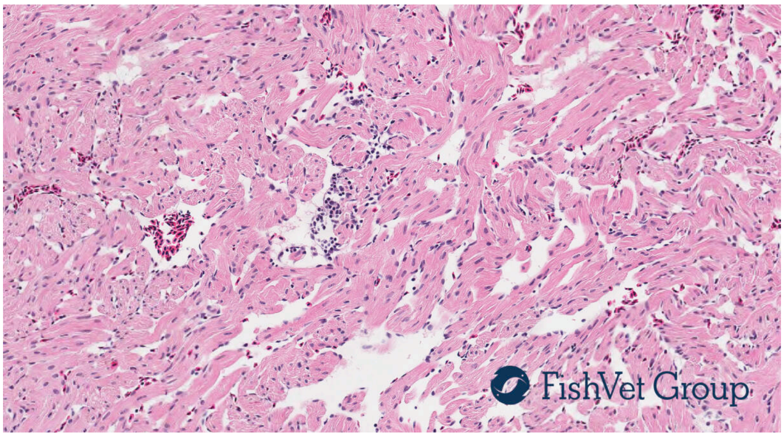

Sample 143. Heart. Spongiosum. Score 0.5

-------------------END OF REPORT-------------------------

For your information

This report should only be redistributed in its full, original version. The report must not be altered without the prior, written approval of Fish Vet Group. The results given in this report are valid only for the samples analysed by Fish Vet Group. The results apply only to the group or individuals the samples originate from. Fish Vet Group is not liable for damage claims or other matters that may arise as a result of, or in connection with, the use of the results given in this report.
