# Supplementary material for: PRV-1 Virulence in Atlantic Salmon Is Affected by Host Genotype
Source: Viruses. 2025 Feb 19;17(2):285. doi: 10.3390/v17020285 (PMC11860446; doi:10.3390/v17020285)
Supplement: Supplementary file 1 [file viruses-17-00285-s001.zip › viruses-3471247-supplementary/viruses-3471247-SM/viruses-3471247-Figure S2.pdf]

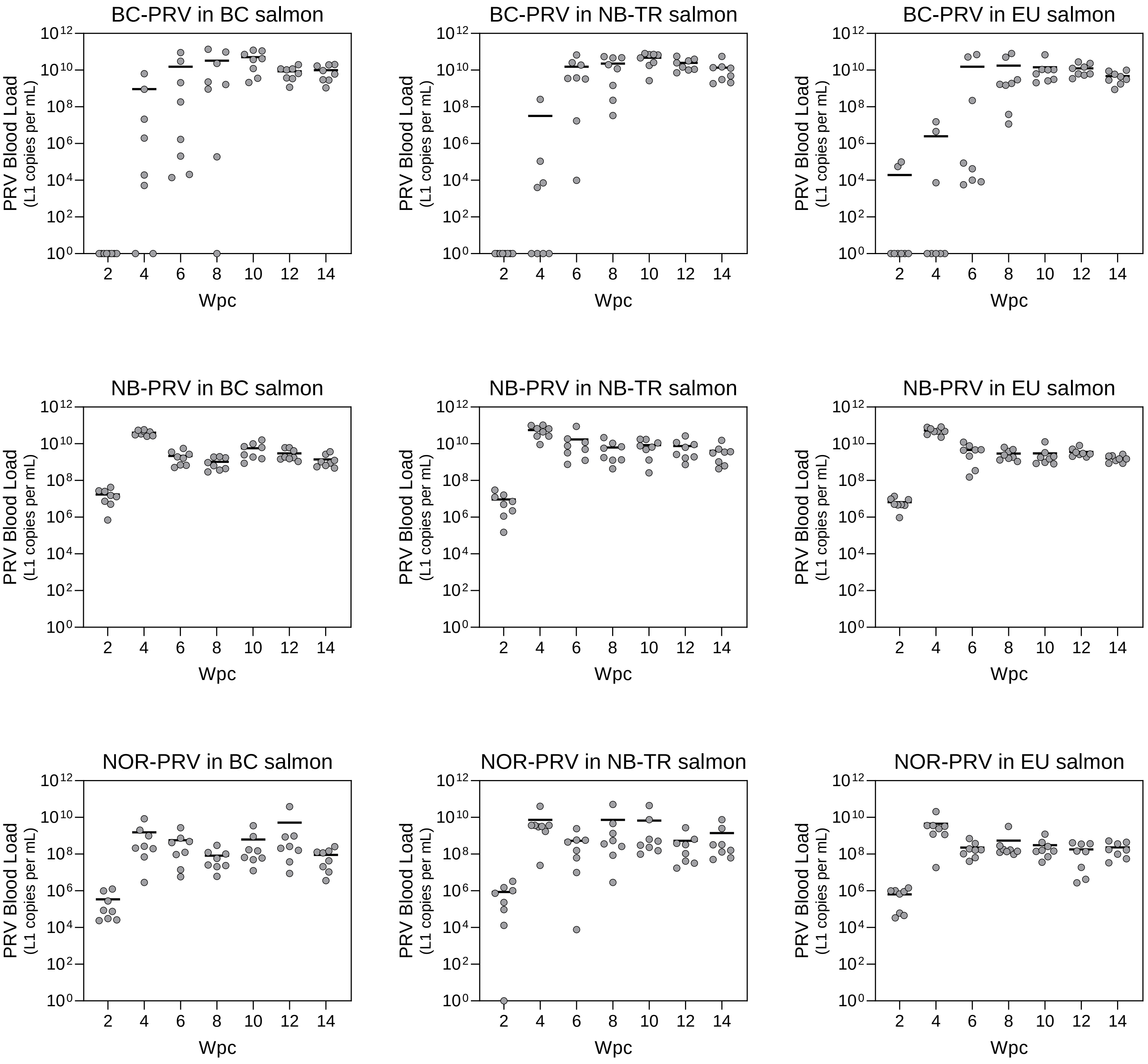

Figure S2. PRV-1 Blood load in side-by-side challenge of BC Mowi-McConnell (BC), New Brunswick Tobique River (NB-TR) and European (EU) Atlantic salmon administered either BC-16-005ND (BC-PRV), NOR-2018NL (NOR-PRV), or NB-2018-128 (NB-PRV) isolates by i.p. injection. Mean (line) and individual (dot) PRV-1 L1 RNA loads measured 2-14 weeks post-challenge (wpc).
